# Computational Insights into the Effect of Noncovalent S···S Interaction on the Excited-State Characteristics of Multiresonant Fluorophore

**DOI:** 10.3390/molecules31122076

**Published:** 2026-06-13

**Authors:** Sunwoo Kang, Taekyung Kim

**Affiliations:** 1 Department of Chemistry, Dankook University, Cheonan-si, 31116, Republic of Korea; 2 Department of Chemical Engineering, Kyung Hee University, Yongin-si 17104, Republic of Korea

**Keywords:** time dependent density functional theory, MR fluorophore, thiophene, vibrationally resolved spectrum, S···S interaction, STEOM-DLPNO-CCSD calculation

## Abstract

The photophysical properties of the designed molecules were investigated by theoretical calculations. The introduction of thiophene units into the **DABNA-1** core reduces both S_1_ and T_1_ energies, whereas the derived ∆*E_ST_* values become larger. As revealed by normal mode analysis for all designed molecules, the designed molecule, including the S···S interaction, exhibits the lowest reorganization energy during the excitation and de-excitation. Vibrationally resolved emission spectra further show that S···S interaction plays a pivotal role in reducing the spectrum width. Comprehensively, it is evident that the S···S interaction is a useful chemical design strategy to suppress the *k_nr_* and enhance the color purity for OLED emitter.

## 1. Introduction

Since the first reported by Hatakeyama et al. [1], the fluorescent chromophore including B and N atoms, which is nominated as the multiresonance (MR) fluorophore, has been paid great attentions since the attractive photophysical features such as the narrow full-width half maximum (FWHM) and the small Stokes shift play a critical role in enhancing the efficiency and color purity of the organic light emitting-diode (OLED) device [2,3,4]. Thanks to these fascinating features, the blue MR fluorophore has been successfully applied in the commercial blue-fluorescent OLED device. Moreover, recent endeavors are tremendously underway to use it as the final emitter of the hyperfluorescent OLED (HF-OLED) devices in the application of the commercial display panel [5,6,7,8,9,10]. This can be explained by the following reasons. (1) Small Stokes shift enables efficient energy transfer through the FRET mechanism from the triplet-harvesting sensitizer to the MR fluorophore. (2) Narrow FWHM significantly contributes to minimizing efficiency loss by means of the micro-cavity effect. Therefore, a superbly efficient and durable HF-OLED device can be implemented, compared to phosphorescent and TADF devices.

Due to the popularity and importance of the MR fluorophore in OLED devices, most studies have focused on expanding the chemical space and understanding the nature of photophysical properties [11,12,13,14,15]. In principle, the root cause of the narrow FWHM and the small Stokes shift is the small displacement of the potential energy surfaces between the singlet ground and excited states (S_0_ and S_1_ states), thereby the displacement of the vibronic states for both states can be minimized. In other words, the effect of the structural changes on the electron-phonon coupling is crucial in determining the FWHM and Stokes shift during the excitation and de-excitation processes.

In chemical and biological systems, sulfur-based attractive interactions play a pivotal role in determining stability, geometry, bioactivity, and folding structure in the protein system [16]. Moreover, thiophene-based organic molecules are widely used in organic field-effect transistor (OFET). In particular, it is noteworthy that closely neighboring thiophene units form a herringbone bulk structure through intermolecular S···S interactions, which has a positive effect on improving the performance in the organic field-effect transistor [17,18,19,20]. In detail, S···S interaction assists in forming a well-packed bulk structure and reduces the structural relaxation via redox reaction due to the interlocking effect. Therefore, S···S interaction not only enhances the orbital overlap but also reduces the reorganization energy, resulting in the fast hole and electron hopping rates [21]. From this perspective, we noted that S···S interaction can be utilized as a chemical tool to suppress structural changes during excitation and de-excitation processes. In addition, the positive effect of the S atom on the TADF property is known as the enhancement of the spin-orbit coupling (SOC) between S_1_ and T_1_ states due to the large effective nuclear charge (SOC ≈ αZ^4^). As a result, the spin conversion yields from T_1_ to S_1_ states can be remarkably enhanced by a fast reverse intersystem crossing rate (*k_rISC_*), enabling the high utilization efficiency of triplet excitons into photons.

**Figure 1:**
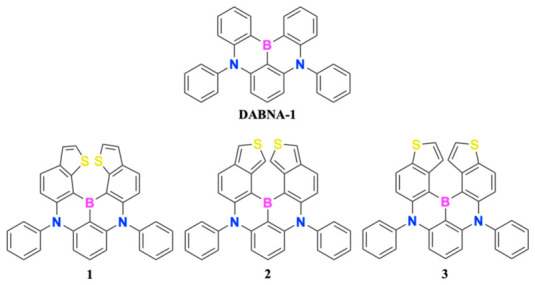
The chemical structures of **DABNA-1** and newly designed molecules (**1**–**3**).

Considering the advantages of the S atom and S···S interaction on molecular properties, we rationally designed three chromophores containing **DABNA-1** core and thiophene moieties to compare and understand the role of the S···S interaction on the essential photophysical properties of the MR fluorophore, as shown Figure 1. Density functional theory (DFT) has become the most powerful tool to understand the nature of molecular properties. In addition, the use of DFT calculations has been expanded as a virtual design tool to find new candidate molecules prior to synthesizing a material [22,23,24]. In the present work, we theoretically investigated the essential photophysical properties to better understand the designed MR fluorophores. We believe that our study will importantly contribute to gaining a new insight into manipulating the excited state parameters and expand the design horizons of MR fluorophores.

## 2. Theory and Computation

Density functional theory (DFT) calculations for the ground state were performed by employing a non-local density functional of Becke’s three-parameterized Lee-Yang-Parr exchange functional (B3LYP) [25,26,27] with Pople’s triple zeta potential with double polarization function (6-311G**), as implemented in the suite of Gaussian 16 program [28]. To understand the excited state properties, time-dependent DFT (TDDFT) calculations in conjunction with Tamm-Dancoff approximation (TDA) [29] were conducted at the same level of theory. All molecular structures in ground and excited states were fully optimized without symmetry constraints, and their thermodynamic stabilities were verified by frequency calculations. In addition, Grimme’s dispersion correction with Bekes–Johnson damping (GD3BJ) was considered to describe the noncovalent S···S interaction in both ground and excited states [30]. According to the previous reports, wave-function methods such as equation-of-motion coupled-cluster single and double (EOM-CCSD) [31], second-order algebraic diagrammatic construction (ADC(2)) [32], spin-component scaling second-order algebraic diagrammatic construction (SCS-ADC(2)) [33], and similarity transformed EOM domain-based local pair natural orbital CCSD (STEOM-DLPNO-CCSD) [34,35,36] should be utilized to accurately describe the nature of the lowest singlet and triplet excited states (S_1_ and T_1_) since the correlation effect must be considered in MR fluorophores. Among them, we recently reported that the single-point STEOM-DLPNO-CCSD calculation at the optimally tuned LC-ωHPBE (LC-ω*HPBE) level of theory provides the quantitative prediction of S_1_ and T_1_ energies [35]. However, the lack of GD3BJ parameters in the LC-ωHPBE functional is a hurdle to computing both excited states. Therefore, the B3LYP functional was utilized as an alternative functional to optimize the molecular structures in the S_1_ and T_1_ states. At the optimized structures of the S_1_ and T_1_ states, STEOM-DLPNO-CCSD calculations with Def2-SVP and their corresponding auxiliary basis sets were further conducted to gain the quantitative S_1_ and T_1_ energies. In addition, the RIJCOSX approximation was utilized to consider the acceleration of the SCF. The scalar-relativistic zero-order regular approximation (ZORA) Hamiltonian was utilized to gain the spin-orbit coupling matrix element (SOCME) between the S_1_ and T_1_ states, as implemented in the suite of ORCA 5.0 program [37]. All SOCME calculations were performed at the B3LYP/TZVP level of theory for the optimized T_1_ state. The reverse intersystem crossing rate (*k_rISC_*) can be obtained by the semi-empirical Marcus theory equation as follows [38].

(1) $$k_{rISC}=\frac{4\pi^{2}{\langle S_{1}\left|H_{SOC}\right|T_{1}\rangle }^{2}}{h}\frac{1}{\sqrt{4\pi k_{b}T\lambda }}exp(-\left(\frac{{\left({\Delta E}_{ST}+\lambda \right)}^{2}}{4\lambda k_{b}T}\right))$$

where <S_1_|H_SOC_|T_1_>, *λ*, *k_B_*, *h*, and T are defined as the spin-orbit coupling constant between S_1_ and T_1_ states, reorganization energy (S_1_ and T_1_ states), Boltzmann constant, Planck constant, and temperature, respectively. Moreover, the radiative decay rate (*k_r_*) during de-excitation can be computed based on Einstein’s spontaneous emission equation [39].

(2) $$k_{r}=\frac{f{\left(E_{emission}\right)}^{2}}{1.449}$$

where *E_emission_* and *f* are defined as the S_1_ energy and oscillator strength. The Huang-Rhys factor (*S*) can be calculated by the following equation,

(3) $$Huang-Rhys\;factor\;(S)=\frac{1}{2}\omega_{i}K_{i}^{2}$$

where $K_{i}$ is the shift vector, and *ω*_i_ is the vibrational frequency of the *i*-th normal vibrational mode. The $K_{i}$ can be defined as a dimensionless displacement vector corresponding to changes in geometries between the initial and final states of the *i*-th normal vibrational mode [40].

The vibrationally resolved emission spectra calculations were performed to gain insight into the S···S interaction effect on the spectra shapes. The combined Franck-Condon/Herzberg-Teller (FCHT) method within the adiabatic Hessian model was adopted to compute the vibrationally-resolved emission spectrum using a half-width at half-maximum (HWHM) value of 680 cm^−1^. In all simulated spectra, the Duschinsky rotation effect was taken into account, and the spectra were generated at 298.15 K.

## 3. Results and Discussion

### 3.1. Structural and Electronic Properties

**Figure 2:**
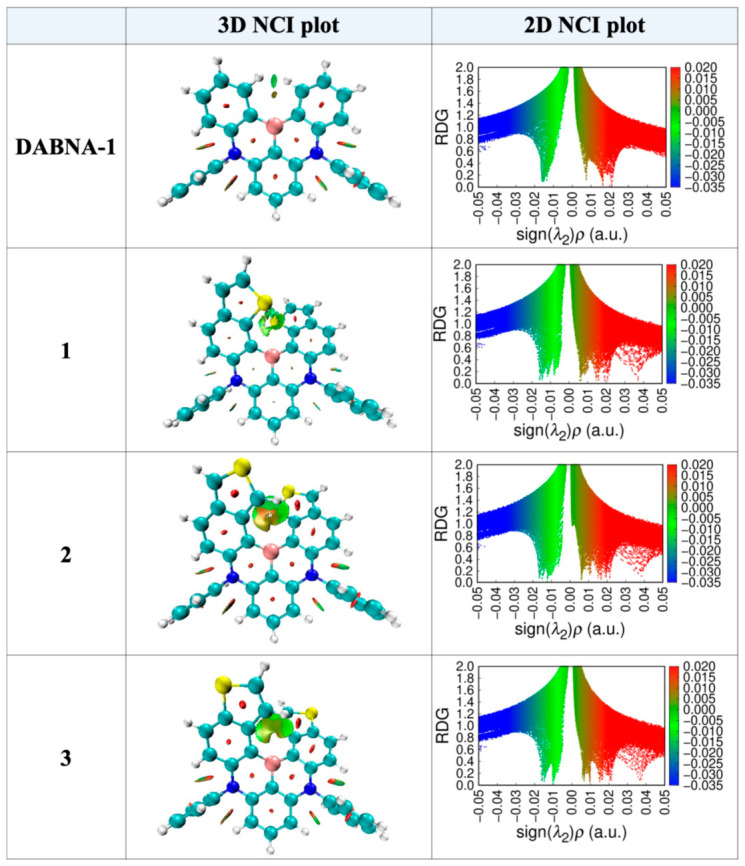
The 3D and 2D NCI plots of **DABNA-1**, **1**, **2**, and **3** molecules. The blue, green, and red colors indicate the strong attractive interaction, weak attractive interaction, and repulsive interaction, respectively.

To clarify the presence of the intramolecular interaction as a function of different positions of S atoms, the analysis and visualization of the noncovalent interaction (NCI) were performed using Multiwfn and VMD programs [41,42]. The 3D NCI plots and 2D NCI plots of reduced density gradient (RDG) vs. sign(λ_2_)*ρ* are shown in Figure 2.

The 3D NCI plot of **DABNA-1** reveals that the intramolecular interaction can be negligible. By introducing the thiophene unit, in the cases of **2** and **3**, the attractive and repulsive intramolecular interactions assigned as π···H attractive and H···H repulsive interactions are remarkably enhanced between the two thiophene moieties. Interestingly, the intramolecular interaction, which can be assigned as an S···S interaction, solely appears in **1**. To support the NCI analysis for **1**, the distance between two S atoms (*d*_S_···_S_) should be compared to the sum of the van der Waals (vdW) radius of two S atoms (3.630 Å). The *d*_S_···_S_ of **1** is calculated to be 3.365 Å, which is remarkably shorter than 3.630 Å. This result is additional evidence for the existence of the attractive S···S interaction in **1**. Furthermore, QTAIM topological analysis for **1** was conducted to clearly explain. A bond critical point (BCP) was identified between the two sulfur atoms with an electron density (ρ(r)) and a positive Laplacian of electron density (∇^2^ρ). Furthermore, the negative sign(λ_2_)ρ value indicates an attractive noncovalent interaction. (See Table S1) Consequently, both NCI and QTAIM analyses consistently demonstrate the existence of an attractive intramolecular S···S interaction. The electronic properties at the optimized S_0_ state are listed in Table 1. Compared to **DABNA-1**, the role of introducing the thiophene unit into the **DABNA-1** core on the electronic properties of **1**–**3** is that the highest occupied molecular orbital (HOMO) energy is destabilized, while the lowest unoccupied molecular orbital (LUMO) energy is stabilized. Correspondingly, the calculated HOMO-LUMO energy gaps of **1**–**3** are smaller than that of **DABNA-1**, expecting the bathochromic shift of both excited state energies.

### 3.2. Excited States Properties

**Figure 3:**
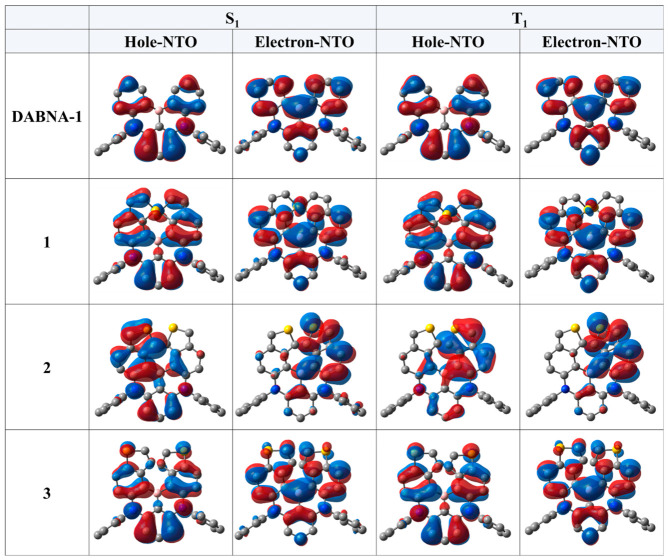
The hole-NTO and electron-NTO of **DABNA-1**, **1**, **2**, and **3** molecules.

The optimized structures and their corresponding natural transition orbitals (NTOs) for the S_1_ and T_1_ states are denoted in Figure 3. Due to the increase in the conjugation length, both NTOs of **1** and **3** spread over the entire molecular structures. In addition, the transition characteristics of these molecules can be assigned as short-range charge transfer, which is similar to those of **DABNA-1**. In contrast, **2** displays distinctly separated spatial distributions of hole and electron NTOs in both S_1_ and T_1_ states. For both S_1_ and T_1_ states, hole-NTOs spread on the main skeleton except one thiophene moiety, while electron-NTOs predominantly lie on the thiophene linked with the phenyl moiety. This result suggests a more pronounced charge-transfer character for the S1 state of **2**, while the T_1_ state retains a localized exciton. The dominant NTO pair contribution of all chromophores is larger than 96% in both excited states (See Table S2). This result indicates that the excited-state characteristic can be interpreted by using the corresponding NTO distributions.

The calculated S_1_ and T_1_ energies of **DABNA-1** and **1**–**3** are listed in Table 2. The calculated S_1_ and T_1_ energies of **1**–**3** at the B3LYP level of theory exhibit the bathochromic shift. Moreover, ∆*E_ST_* values of these molecules are increased compared to **DABNA-1**. As mentioned in the theory and computation, predicting the S_1_ and T_1_ energies of the MR-type chromophore is essentially considered the electron-electron correlation effect. In other words, the TDDFT result is inadequate to obtain the quantitative S_1_ and T_1_ energies in the MR-type chromophore. Therefore, STEOM-DLPNO-CCSD results should be considered quantitatively. In the experiment, the observed S_1_ and T_1_ energies of **DABNA-1** are 2.781 eV and 2.620 eV, while the theoretically calculated S_1_ and T_1_ energies are 2.781 eV and 2.721 eV. This result indicates that the choice of our theoretical methodologies guarantees the quantitative prediction of the photophysical properties of the MR chromophore. By introducing the thiophene unit into **DABNA-1**, the calculated S_1_ and T_1_ energies of **1**–**3** are smaller than those of **DABNA-1**, which is qualitatively consistent with TDDFT calculations. Although the emission energies of **1**–**3** slightly shifted to lower energies, the emission colors of **1**–**3** belong to the blue color region. This result indicates that the designed molecules can be potentially utilized as a blue emitter. Based on the obtained S_1_ and T_1_ energies, the derived ∆*E_ST_* values of **1**–**3** are relatively increased. Among them, it is noteworthy that the derived ∆*E_ST_* of **2** is 1.211 eV, which is too large to activate the spin-flip transition from T_1_ to S_1_ states, indicating the pure fluorophore without delayed fluorescence emission. On the other hand, ∆*E_ST_* values of **1** and **3** are not yet sufficient to precisely judge the emission mechanism. Therefore, further analyses for **1** and **3** are needed to accurately confirm whether they show delayed emission. By comparing **1** and **3**, the effect of S···S interaction on the S_1_ energy is negligible. In contrast, S···S interaction significantly affects the T_1_ energy, resulting in a relatively high T_1_ energy for **1**.

To address the emission mechanism of designed molecules, several parameters related to exciton kinetics were computed and listed in Table 3. The oscillator strength (*f*) can be used to judge the transition characteristic as well as predict the radiative decay rate. Compared to **DABNA-1**, the calculated *f* of **1** is slightly increased, while that of **3** is similar. On the other hand, the calculated *f* of **2** is significantly decreased. According to the definition of *f*, the overlap of molecular orbitals contributing to the transition is one of the key determinants and proportional to *f.* The spatial distributions of both NTOs suggest a more pronounced charge-transfer character in **2** compared with the other chromophores. The theoretically predicted radiative decay rate (*k_r_*) of **DABNA-1** is 5.02 × 10^7^ s^−1^, which is decreased to 4.94 × 10^7^ s^−1^, 1.18 × 10^7^ s^−1^, and 4.50 × 10^7^ s^−1^ in **1**–**3**. Specifically, the theoretically predicted *k_r_* of **2** is remarkably reduced due to the small *f.* As listed in Table 3, the calculated H_SOC_ values of **DABNA-1**, **1**, **2**, and **3** are 0.017 cm^−1^, 0.081 cm^−1^, 0.458 cm^−1^, and 0.098 cm^−1^, respectively. By introducing the thiophene units into the **DABNA-1** core, H_SOC_ values are generally increased due to the heavy atom effect of the S atom. However, the magnitude of H_SOC_ is not directly correlated with the presence of the intramolecular S···S interaction. In particular, **2** exhibits the remarkably enhanced H_SOC_ value despite the absence of the intramolecular S···S interaction. This can be understood by means of the EI-Sayed rule because the S_1_ and T_1_ states reveal the different transition characters, which can be assigned to the ^1^CT and ^3^LE states [43]. Therefore, the large H_SOC_ value of **2** mainly originates from the nature of the electronic structures of both excited states rather than intramolecular S···S interaction. To calculate the reverse intersystem crossing rate (*k_rISC_*), the reorganization energy between S_1_ and T_1_ states (*λ*) is assumed to be 0.16 eV, which is a representative value that concerns the medium-induced relaxation, as established in previous studies [34,44,45]. Therefore, the calculated *k_rISC_* value should be considered as a qualitative indicator rather than a quantitative rate constant. Given the H_SOC_ and *λ*, the theoretically predicted *k_rISC_* values of **DABNA-1**, **1**, **2**, and **3** are 1.02 × 10^4^ s^−1^, 2.63 × 10^2^ s^−1^, 0 s^−1^, and 1.75 × 10^0^ s^−1^, respectively. Compared to **DABNA-1**, the theoretically predicted *k_rISC_* values of **1**–**3** are extremely reduced, despite the strong H_SOC_. Therefore, these results suggest that ∆*E_ST_* may play a more important role than H_SOC_ in determining the theoretically predicted *k_rISC_* values in newly designed molecules. Furthermore, it is noteworthy that the designed **1**–**3** molecules are not expected to appear the TADF characteristic due to the unfavorable spin-flip transition.

### 3.3. Normal Mode Analysis and Vibrationally Resolved Spectra

**Figure 4:**
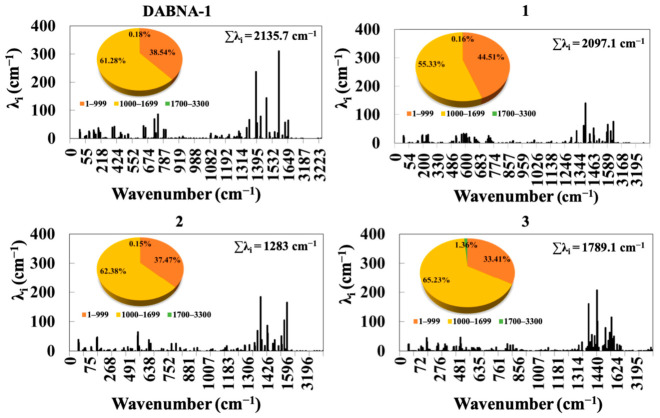
The plotted NM analyses of **DABNA-1**, **1**, **2**, and **3** between S_0_ and S_1_ states.

The normal-mode (NM) analyses based on the *S* were plotted in Figure 4. Moreover, the inset 3D pie charts show the vibrational mode contributions to ∑*λ_i_* in the ranges of 1–999 cm^−1^, 1000–1699 cm^−1^, and 1700–3300 cm^−1^, which represent the C-H out-of-plane bending, C-H in-plane bending/stretching, and C-H stretching vibration modes. It can be seen that the effect of introducing the thiophene unit into the **DABNA-1** unit on the ∑*λ_i_* is to suppress the structural relaxation, thereby mitigating the non-radiative decay process during the S_1_ ⟶ S_0_ transition. In detail, the most contributive vibration modes to ∑*λ_i_* between S_0_ and S_1_ states are 1351.11 cm^−1^, 1498.35 cm^−1^, and 1626.27 cm^−1^, which corresponds to the C-H in-plane bending mode and mixing of C-C stretching and C-H in-plane bending modes in **DABNA-1**. (See Figure S1) These vibration modes are less activated in **1**–**3** due to the presence of intramolecular S···S and π···H interactions. Moreover, (∑*λ_i_*)s of **1**–**3** show the larger value in the order of **1** < **2** < **3**, suggesting that S···S interaction is the most significant chemical design strategy to hinder structural changes during the excitation and de-excitation. By considering the interrelationship between structural relaxation and non-radiative decay rate (*k_nr_*), it is also expected that S···S interaction plays an important role in achieving the good quantum efficiency by mitigating the activation of *k_nr_*. To more clearly understand the *k_nr_* behaviors of designed molecules, we have computed *k_nr_* based on the $k_{nr}\propto \;\sum_{i}S_{i}\omega_{i}e^{-S_{i}}$ equation. The theoretically predicted *k_nr_* values of **DABNA-1**, **1**, **2**, and **3** are 0.1109, 0.0737, 0.2975, and 0.0954, respectively. In the experiment, *k_nr_*(s) of **DABNA-1** is derived as 1.76 × 10^7^ s*^−^*^1^. To match the exponent of the *k_nr_* in the experiment, the coefficient derived from **DABNA-1** (1.587 × 10^8^) was equally multiplied by **1**–**3**. As a result, the theoretically predicted *k_nr_* values of **1**–**3** are rewritten as 1.17 × 10^7^ s*^−^*^1^, 4.72 × 10^7^ s*^−^*^1^, and 1.51 × 10^7^ s*^−^*^1^, respectively. It is evident that the positive role of S···S interaction on the mitigation of *k_nr_*.

To clearly identify the effect of the S···S interaction on the vibrational modes, Huang-Rhys factor analyses were further performed for **1**–**3**. As shown in Figure S3, the presence of the S···S interaction clearly suppressed the thiophene-related out-of-plane bending and in-plane bending in the low-frequency region. Particularly, 2 exhibits strong activation of the 21.11 cm^−1^ and 71.41 cm^−1^ vibration modes with contributions of 18.4% and 22.8%. Likewise, 3 displays a large contribution (49.6%) of 21.39 cm^−1^ vibration mode, which is strongly activated relative to **1**. These results demonstrate that the intramolecular S···S interaction imposes conformational restrictions, thereby contributing to the reduction of the reorganization energy and electron-phonon coupling.

**Figure 5:**
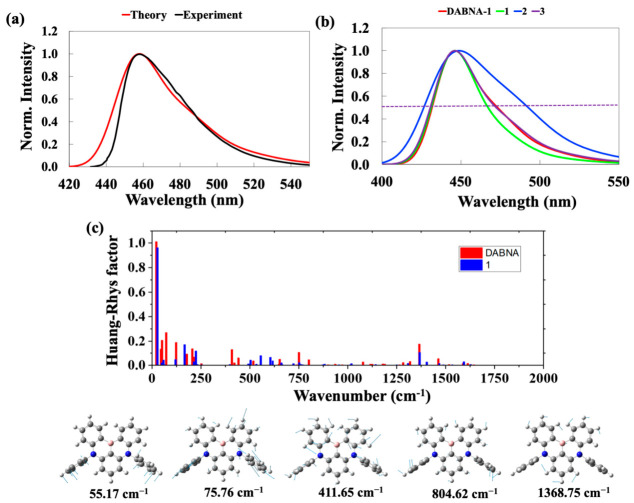
(**a**) The spectral shapes of **DABNA-1** in theory and experiment. (**b**) The simulated spectral shapes of **DABNA-1**, **1**, **2**, and **3** with HWHM = 680cm^−1^. (**c**) The *S* plot for **DABNA-1** and **1**. The representative vibrational modes with large *S* values are illustrated.

Lastly, we conducted vibrationally resolved spectrum calculations to understand the effect of the S···S interaction on the spectrum shape. Prior to analyzing the emission spectra, the spectral similarity of **DABNA-1** in theory and experiment should be confirmed. Therefore, we compared the theoretical and experimental emission spectra of **DABNA-1**. The maximum peak (*λ_max_*) of the theory was blue-shifted to match with the experiment. As shown in Figure 5a, the theoretically predicted spectrum shape seems to be well-consistent with experiment. Based on the theoretical spectrum of **DABNA-1**, we further compared the emission spectra of **1**–**3,** which are simulated under the same theoretical conditions. Similarly, *λ_max_* values in **1**–**3** were shifted to match the *λ_max_* of **DABNA-1** in order to clearly compare spectral shape and width. The unique emission spectra for each complex are depicted in Figure S2. As shown in Figure 5b, the spectrum shape of **3** is quite similar to that of **DABNA-1**; however, the spectral shapes of **1** and **2** exhibit significant changes. In detail, the spectrum width of **2** is wider than that of **DABNA-1**. On the contrary, it is noteworthy that **1** exhibits a narrower spectrum shape than **DABNA-1**. The calculated FWHMs of **DABNA-1**, **1**, **2**, and **3** are 41.3 nm (2039 cm^−1^), 35.8 nm (1768 cm^−1^), 65.96 nm (3257 cm^−1^), and 41.5 nm (2049 cm^−1^), respectively. Based on the simulated emission spectra of these complexes, a wider FWHM is expected in the order of **2** > **3** ≈ **DABNA-1** > **1**. The spectra with their own emission wavelengths are depicted in Figure S2. To understand the root cause of the change in the spectrum width, the Huang-Rhys factors (*S*) between the S_0_ and S_1_ states were analyzed. Due to the similarity in spectrum shape with **DABNA-1**, we will not discuss the result of *S* for **3**. As shown in Figure S3, it is estimated that the broader spectrum of **2** is mainly attributed to the strong activations of C-H/S out-of-plane bending modes at 21.11 cm^−1^ and 71.41 cm^−1^. On the other hand, for **DABNA-1** and **1**, Figure 5c clearly shows that the in-plane C-H bending modes and out-of-plane C-H bending modes are significantly suppressed due to the presence of S···S interaction. Specifically, the representative vibrational modes contributing to the spectrum width are analyzed 55.17 cm^−1^, 75.76 cm^−1^, 411.65 cm^−1^, 804.62 cm^−1^, and 1368.75 cm^−1^, respectively, which correspond to the in-plane C-H bending and out-of-plane C-H bending modes. Therefore, S values associated with the vibrational modes are significantly reduced in **1** due to the presence of S···S interaction. Upon the de-excitation process from S_1_ to S_0_ states, the suppression of these specific vibrational modes associated with structural changes reduces the reorganization energy and weakens the electron-phonon coupling in the presence of S···S interaction. Consequently, the activation of vibrational modes contributing to the relatively broad-spectrum width can be effectively suppressed, resulting in a narrower spectrum width. From this result, we propose that the S···S interaction might be utilized as a chemical design strategy to improve the color purity of emissive materials in OLED devices.

## 4. Conclusions

In this study, we designed MR chromophores including thiophene units that are theoretically investigated to understand the photophysical properties. From analyses of NCI and *d*_S_···_S_, we confirm the presence of S···S interaction in **1**. The calculated S_1_ and T_1_ energies are decremented by introducing the thiophene units into **DABNA-1**, but the derived ∆*E_ST_* values are relatively increased. The calculated *k_r_* values of **1**–**3** are computed to be 10^7^ s^−1^ order, which is similar to **DABNA-1**. Although Hsoc values are relatively enhanced due to the heavy atom effect, the computed *k_rISC_* values are remarkably slowed by means of the large ∆*E_ST_*. Hence, the non-TADF characteristics are expected in **1**–**3**. Interestingly, it was found that the S···S interaction simultaneously reduces the reorganization energy and spectrum width due to the suppression of vibration-coupled structural relaxation. From our results, we propose that the utilization of S···S interaction is a promising computational design strategy to simultaneously improve the quantum efficiency and color purity of the emitter in achieving a highly efficient OLED device.

## Figures and Tables

**Table 1:** The structural parameter and electronic properties of **DABNA-1** and newly designed molecules.

	*d*_S_···_S_ (Å)	HOMO (eV)	LUMO (eV)	H-L Gap (eV)
**DABNA-1**	-	−4.996	−1.364	3.632
**1**	3.365	−4.900	−1.466	3.434
**2**	4.636	−4.802	−1.546	3.255
**3**	6.627	−4.971	−1.547	3.425

**Table 2:** The computed S_1_, T_1_, and ∆*E_ST_* at the B3LYP/6-311G** level of theory and their corrected energies at the STEOM-DLPNO-CCSD/SVP level of theory.

	B3LYP			STEOM-DLPNO-CCSD		
	S_1_ (eV)	T_1_ (eV)	∆*E_ST_* (eV)	S_1_ (eV)	T_1_ (eV)	∆*E_ST_* (eV)
**DABNA-1**	2.915	2.509	0.406	2.781	2.721	0.060
**1**	2.794	2.327	0.467	2.633	2.394	0.239
**2**	2.237	1.613	0.624	2.618	1.407	1.211
**3**	2.743	2.324	0.419	2.634	2.296	0.338

**Table 3:** The oscillator strength (*f*), spin-orbit coupling constants, and derived exciton dynamics parameters. All theoretically predicted *k_nr_* values are empirically manipulated based on the experimental value of **DABNA-1**.

	*f*	H_SOC_ (cm^−1^)	*k_r_* (s^−1^)	*k_rISC_* (s^−1^)	*k_nr_* (s^−1^)
**DABNA-1**	0.1495	0.017	5.02 × 10^7^	1.02 × 10^4^	1.76 × 10^7^
**1**	0.1643	0.081	4.94 × 10^7^	2.63 × 10^2^	1.17 × 10^7^
**2**	0.0395	0.458	1.18 × 10^7^	≈0	4.72 × 10^7^
**3**	0.1496	0.098	4.50 × 10^7^	1.75 × 10^0^	1.51 × 10^7^

## Data Availability

The original contributions presented in this study are included in the article/Supplementary Material. Further inquiries can be directed to the corresponding authors.

## Supplementary Materials

The following supporting information can be downloaded at: https://www.mdpi.com/article/10.3390/molecules31122076/s1. Figure S1. The representative vibration modes of DABNA-1 contributing to ∑ *λ_i_*. Figure S2. The vibrationally resolved spectra of **DABNA-1**, **1**, **2**, and **3** without spectral shift. Figure S3. The comparison of Huang-Rhys factor plots for 1/2 and 1/3. Table S1. QTAIM parameters for the S···S interaction in **1**. Table S2. The calculated pair contribution of dominant NTOs to the S_1_ state and the T_1_ state. Table S3. The computed spin-orbit coupling element matrix values of **DABNA-1**, **1**, **2**, and **3**. Table S4. The screen shots of STEOM-DLPNO-CCSD calculations for the S_1_ and the T_1_ state. Table S5. The raw data of reorganization energy for **DABNA-1**, **1**, **2**, and **3**. Table S6. Mode-specific reorganization-energy contributions. Table S7. The cartesian coordinates of **1**, **2**, and **3** in the S_1_ state and T_1_ state.
