# Supplementary material for: Computational Insights into the Effect of Noncovalent S···S Interaction on the Excited-State Characteristics of Multiresonant Fluorophore
Source: Molecules. 2026 Jun 13;31(12):2076. doi: 10.3390/molecules31122076 (PMC13304540; doi:10.3390/molecules31122076)
Supplement: Supplementary file 1 [file molecules-31-02076-s001.zip › molecules-4359644-supplementary.pdf]

Computational Insights into the Effect of Noncovalent S···S Interaction on the Excited-State Characteristics of Multiresonant Fluorophore

Sunwoo Kang [1,*] and Taekyung Kim [2,*]

[1] Department of Chemistry, Dankook University, Cheonan-si, 31116, Republic of Korea
[2] Department of Chemical Engineering, Kyung Hee University, Yongin-si 17104, Republic of Korea
* Correspondence: sunwoo.kang@dankook.ac.kr (S.K.); taekyung.kim@khu.ac.kr (T.K.)

Figure S1. The representative vibration modes of **DABNA-1** contributing to $\sum \lambda_i$.

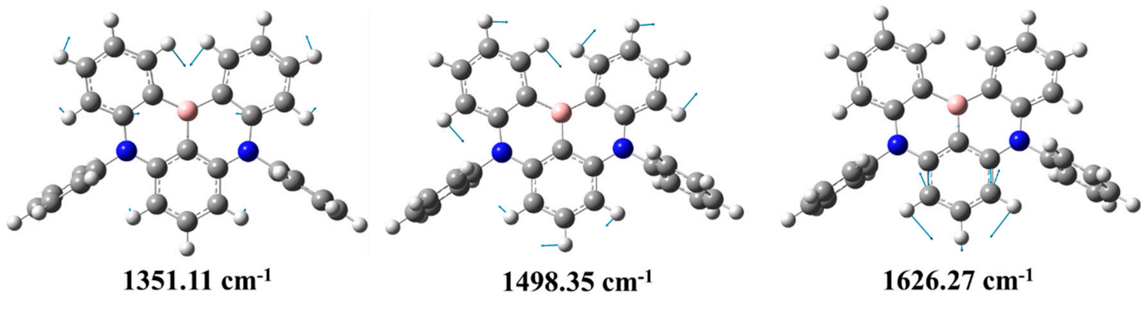

Figure S2. The vibrationally resolved spectra of **DABNA-1**, **1**, **2**, and **3** without spectral shift.

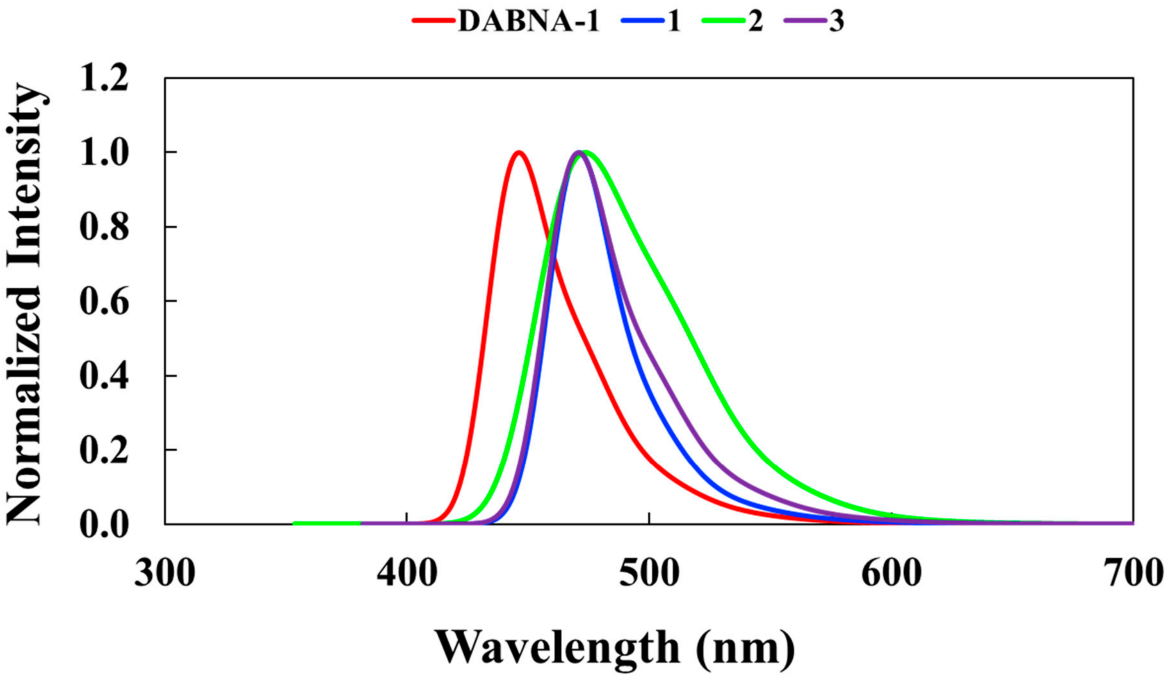

Figure S3. The comparison of Huang-Rhys factor plots for **1**/**2** and **1**/**3**.

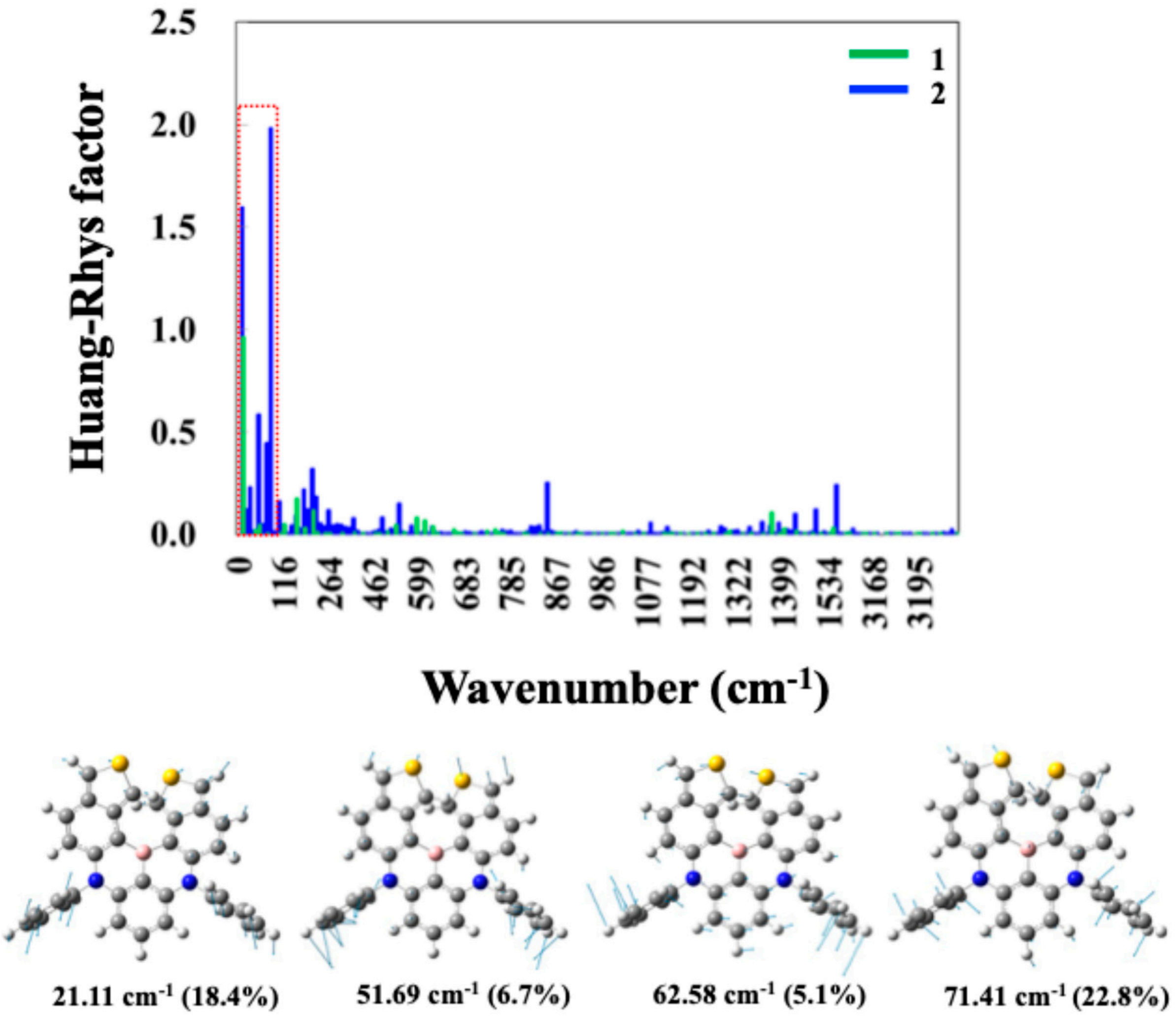

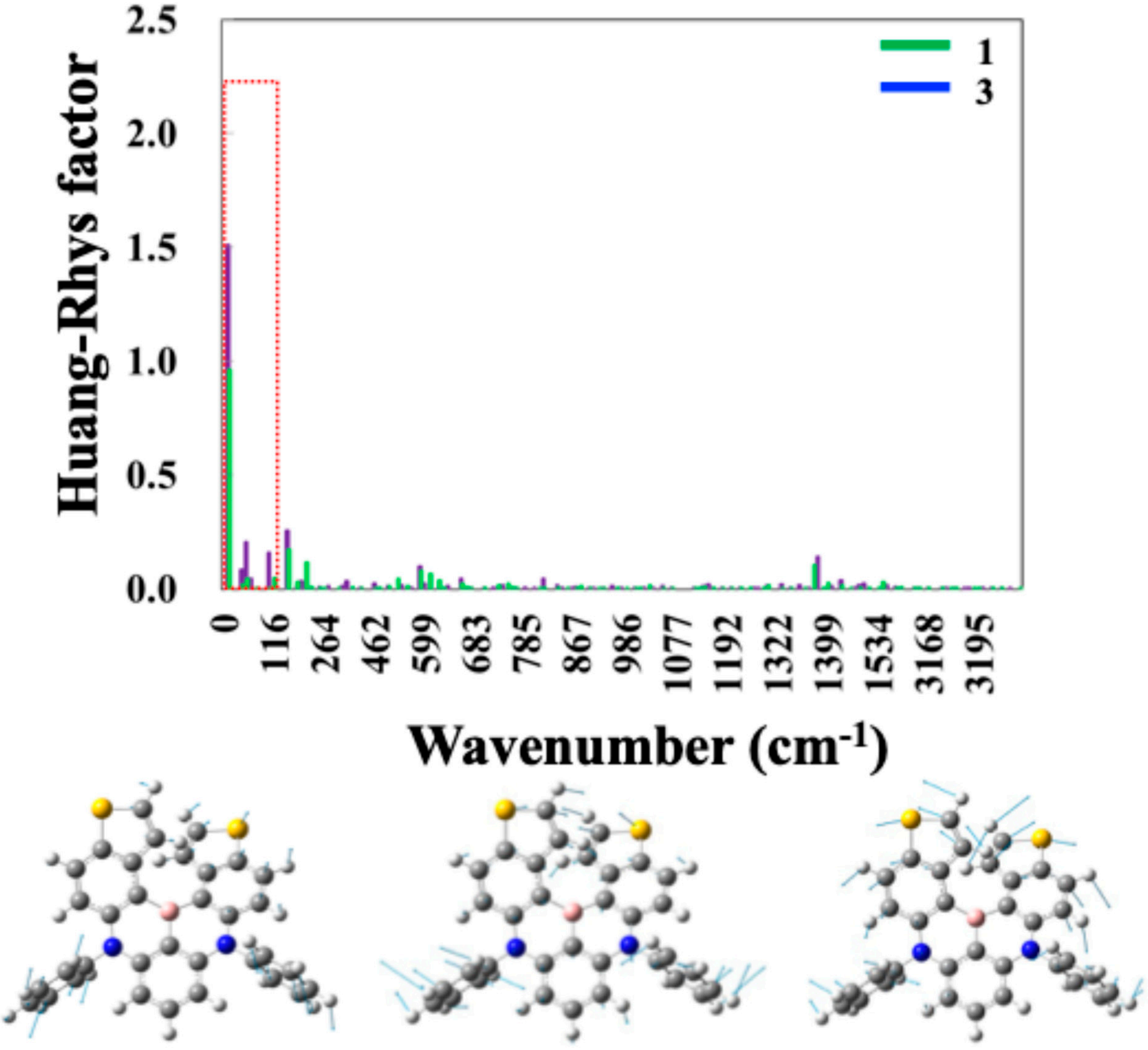

21.39 cm^{-1} (49.6%)

53.11 cm^{-1} (6.7%)

78.83 cm^{-1} (5.2%)

Table S1. QTAIM parameters for the S···S interaction in **1**.

Material	$\rho(r)$	$\nabla^2\rho(r)$	H(r)	$sign(\lambda_2)\rho$	Ellipticity (ε)
1	0.0118	0.0369	0.0011	-0.0118	0.237

Table S2. The calculated pair contribution of dominant NTOs to the S_1 state and the T_1 state.

Materials	Pair contribution of dominant NTOs	
	S_1	T_1
DABNA-1	0.97126	0.98382
1	0.96537	0.97528
2	0.99052	0.97821
3	0.97142	0.97575

Table S3. The computed spin-orbit coupling element matrix values of DABNA-1, 1, 2, and 3.

	SOCME (cm^{-1})		
	X	Y	Z
DABNA-1	0.00	0.03	0.00
1	0.00	0.14	0.00
2	0.56	0.47	0.31
3	0.00	0.17	0.00

Table S4. The screen shots of STEOM-DLPNO-CCSD calculations for the S_1 and the T_1 state.

	S_1	T_1
DABNA-1	IROOT= 1: 0.102189 au 2.781 eV 22427.8 cm**-1<br>Amplitude Excitation<br>-0.112108 108 -> 112<br>-0.139688 108 -> 115<br>0.934349 109 -> 110<br>-0.123361 109 -> 111<br>0.121900 109 -> 118<br>Ground state amplitude: -0.000023<br><br>Percentage Active Character 98.55	IROOT= 1: 0.099990 au 2.721 eV 21945.2 cm**-1<br>Amplitude Excitation<br>0.945244 109 -> 110<br>0.147556 109 -> 118<br>Ground state amplitude: 0.000000<br><br>Percentage Active Character 98.87
1	IROOT= 1: 0.096775 au 2.633 eV 21239.7 cm**-1<br>Amplitude Excitation<br>-0.149001 135 -> 139<br>-0.107629 136 -> 145<br>0.934902 137 -> 138<br>Ground state amplitude: -0.000033<br><br>Percentage Active Character 98.25	IROOT= 1: 0.087989 au 2.394 eV 19311.4 cm**-1<br>Amplitude Excitation<br>0.121393 127 -> 138<br>0.129341 134 -> 138<br>-0.227854 136 -> 139<br>0.889552 137 -> 138<br>-0.122250 137 -> 144<br>Ground state amplitude: 0.000000<br><br>Percentage Active Character 98.45
2	IROOT= 1: 0.096193 au 2.618 eV 21112.0 cm**-1<br>Amplitude Excitation<br>-0.116823 135 -> 138<br>-0.272890 136 -> 138<br>0.793574 137 -> 138<br>0.352868 137 -> 139<br>0.207608 137 -> 140<br>0.141538 137 -> 144<br>0.107408 137 -> 146<br>Ground state amplitude: -0.022255<br><br>Percentage Active Character 98.08	IROOT= 1: 0.051721 au 1.407 eV 11351.4 cm**-1<br>Amplitude Excitation<br>0.205599 135 -> 138<br>0.340310 136 -> 138<br>-0.848415 137 -> 138<br>Ground state amplitude: 0.000000<br><br>Percentage Active Character 98.49
3	IROOT= 1: 0.096782 au 2.634 eV 21241.3 cm**-1<br>Amplitude Excitation<br>0.115898 135 -> 139<br>0.135090 136 -> 143<br>0.945427 137 -> 138<br>Ground state amplitude: -0.000023<br><br>Percentage Active Character 98.62	IROOT= 1: 0.084367 au 2.296 eV 18516.4 cm**-1<br>Amplitude Excitation<br>0.108636 131 -> 138<br>-0.162647 134 -> 138<br>-0.190144 135 -> 139<br>0.902685 137 -> 138<br>0.171883 137 -> 145<br>Ground state amplitude: 0.000000<br><br>Percentage Active Character 98.82

Table S5. The raw data of reorganization energy for **DABNA-1**, **1**, **2**, and **3**.

DABNA-1

Frequency (cm^{-1})	λ_1 (cm^{-1}) ($S_0 \rightarrow S_1$)	Frequency (cm^{-1})	λ_2 (cm^{-1}) ($S_1 \rightarrow S_0$)	λ_{sum} (cm^{-1})	Contribution (%)
25.00	31.50	21.00	21.40	52.90	2.48
25.00	0.00	25.00	0.00	0.00	0.00
49.00	0.00	38.00	0.00	0.00	0.00
50.00	0.00	47.00	6.50	6.50	0.30
55.00	9.20	53.00	10.00	19.20	0.90
73.00	0.00	73.00	0.00	0.00	0.00
76.00	26.00	77.00	21.10	47.10	2.21
85.00	0.00	79.00	0.00	0.00	0.00
92.00	0.00	96.00	0.00	0.00	0.00
127.00	29.60	123.00	22.50	52.10	2.44
182.00	7.40	181.00	14.40	21.80	1.02
193.00	0.00	190.00	0.00	0.00	0.00
210.00	37.30	207.00	29.30	66.60	3.12
218.00	0.60	214.00	21.20	21.80	1.02
218.00	0.00	215.00	0.00	0.00	0.00
255.00	1.40	245.00	0.00	1.40	0.07
256.00	0.00	252.00	2.90	2.90	0.14
270.00	1.90	270.00	1.10	3.00	0.14
285.00	0.00	286.00	0.00	0.00	0.00
348.00	0.00	343.00	0.00	0.00	0.00
365.00	0.00	350.00	0.00	0.00	0.00
403.00	38.90	380.00	0.00	38.90	1.82
412.00	0.00	395.00	42.40	42.40	1.99
422.00	1.70	418.00	0.00	1.70	0.08
424.00	0.00	418.00	0.10	0.10	0.00
424.00	0.80	421.00	3.90	4.70	0.22
445.00	0.00	437.00	21.00	21.00	0.98
446.00	13.90	443.00	0.00	13.90	0.65
471.00	0.00	466.00	0.00	0.00	0.00
479.00	9.70	467.00	1.60	11.30	0.53
505.00	0.00	490.00	0.00	0.00	0.00
522.00	15.30	519.00	15.10	30.40	1.42

537.00	0.00	533.00	0.00	0.00	0.00
550.00	0.10	541.00	0.00	0.10	0.00
552.00	0.00	553.00	0.00	0.00	0.00
627.00	0.00	578.00	0.00	0.00	0.00
629.00	0.10	615.00	0.00	0.10	0.00
634.00	0.00	621.00	1.10	1.10	0.05
635.00	0.00	630.00	0.00	0.00	0.00
639.00	0.00	631.00	0.00	0.00	0.00
641.00	0.00	637.00	0.00	0.00	0.00
652.00	44.70	646.00	5.20	49.90	2.34
656.00	0.50	648.00	38.40	38.90	1.82
665.00	0.00	659.00	0.00	0.00	0.00
674.00	1.00	662.00	0.30	1.30	0.06
717.00	0.00	670.00	0.00	0.00	0.00
717.00	1.60	715.00	0.00	1.60	0.07
743.00	0.00	716.00	2.30	2.30	0.11
750.00	69.00	722.00	0.10	69.10	3.24
752.00	19.60	731.00	0.00	19.60	0.92
754.00	0.00	742.00	88.40	88.40	4.14
768.00	0.00	746.00	0.00	0.00	0.00
774.00	0.40	750.00	0.00	0.40	0.02
777.00	0.00	755.00	1.00	1.00	0.05
787.00	32.30	779.00	0.00	32.30	1.51
805.00	0.00	781.00	31.00	31.00	1.45
811.00	0.00	800.00	0.00	0.00	0.00
854.00	0.20	841.00	0.00	0.20	0.01
856.00	0.00	846.00	0.90	0.90	0.04
861.00	1.10	852.00	0.10	1.20	0.06
870.00	0.00	852.00	0.00	0.00	0.00
871.00	0.00	867.00	1.30	1.30	0.06
903.00	0.70	892.00	0.80	1.50	0.07
905.00	0.00	892.00	0.00	0.00	0.00
919.00	4.30	895.00	0.00	4.30	0.20
935.00	0.00	899.00	0.20	0.20	0.01
940.00	2.40	908.00	7.50	9.90	0.46
962.00	2.00	920.00	0.00	2.00	0.09
962.00	0.00	933.00	1.10	1.10	0.05
965.00	0.00	948.00	0.00	0.00	0.00
974.00	0.30	948.00	0.00	0.30	0.01
976.00	0.00	954.00	1.70	1.70	0.08
986.00	0.00	964.00	0.00	0.00	0.00
986.00	0.00	971.00	1.20	1.20	0.06
988.00	0.00	980.00	0.00	0.00	0.00
998.00	0.40	980.00	0.00	0.40	0.02
1007.00	0.00	1006.00	0.00	0.00	0.00
1007.00	0.20	1006.00	0.30	0.50	0.02
1025.00	0.00	1022.00	0.00	0.00	0.00
1025.00	1.20	1023.00	1.20	2.40	0.11
1048.00	0.00	1044.00	0.00	0.00	0.00
1050.00	0.10	1050.00	0.20	0.30	0.01
1065.00	0.00	1057.00	0.00	0.00	0.00

1080.00	0.00	1075.00	0.00	0.00	0.00
1082.00	10.60	1077.00	17.60	28.20	1.32
1098.00	0.00	1097.00	0.00	0.00	0.00
1098.00	0.00	1097.00	0.00	0.00	0.00
1119.00	4.00	1112.00	9.70	13.70	0.64
1129.00	2.00	1127.00	6.90	8.90	0.42
1146.00	0.00	1129.00	0.00	0.00	0.00
1183.00	3.80	1152.00	0.00	3.80	0.18
1183.00	0.00	1167.00	11.00	11.00	0.52
1183.00	0.40	1179.00	0.00	0.40	0.02
1189.00	0.00	1183.00	0.00	0.00	0.00
1192.00	0.20	1183.00	1.70	1.90	0.09
1193.00	0.00	1183.00	5.90	5.90	0.28
1197.00	13.60	1191.00	0.50	14.10	0.66
1210.00	0.00	1191.00	0.00	0.00	0.00
1230.00	0.00	1199.00	0.00	0.00	0.00
1232.00	0.50	1200.00	6.00	6.50	0.30
1268.00	0.00	1223.00	3.20	3.20	0.15
1273.00	6.80	1224.00	0.00	6.80	0.32
1287.00	0.00	1265.00	25.40	25.40	1.19
1302.00	17.90	1273.00	0.00	17.90	0.84
1314.00	4.30	1304.00	0.00	4.30	0.20
1320.00	0.00	1313.00	0.00	0.00	0.00
1322.00	0.00	1314.00	1.00	1.00	0.05
1322.00	0.00	1319.00	38.30	38.30	1.79
1340.00	0.00	1324.00	0.00	0.00	0.00
1342.00	67.20	1324.00	0.20	67.40	3.16
1347.00	0.00	1341.00	0.00	0.00	0.00
1347.00	0.00	1347.00	0.00	0.00	0.00
1361.00	0.00	1347.00	0.10	0.10	0.00
1369.00	0.00	1351.00	237.10	237.10	11.11
1395.00	55.00	1374.00	0.00	55.00	2.58
1462.00	0.00	1429.00	0.00	0.00	0.00
1467.00	7.30	1455.00	78.60	85.90	4.02
1481.00	0.00	1460.00	0.20	0.20	0.01
1484.00	0.00	1463.00	0.00	0.00	0.00
1484.00	0.00	1482.00	0.00	0.00	0.00
1498.00	143.80	1482.00	0.00	143.80	6.74
1514.00	0.00	1482.00	0.00	0.00	0.00
1521.00	20.90	1517.00	7.10	28.00	1.31
1530.00	0.00	1527.00	0.00	0.00	0.00
1532.00	1.00	1529.00	0.90	1.90	0.09
1592.00	0.00	1531.00	23.40	23.40	1.10
1600.00	1.90	1540.00	0.00	1.90	0.09
1617.00	0.00	1565.00	19.40	19.40	0.91
1626.00	311.10	1566.00	0.00	311.10	14.57
1632.00	0.00	1598.00	0.00	0.00	0.00
1632.00	0.00	1608.00	11.30	11.30	0.53
1641.00	0.00	1624.00	0.00	0.00	0.00
1642.00	57.30	1624.00	0.10	57.40	2.69
1648.00	0.00	1634.00	6.10	6.10	0.29

1649.00	64.30	1634.00	0.00	64.30	3.01
3164.00	0.00	3158.00	0.00	0.00	0.00
3164.00	1.10	3160.00	0.60	1.70	0.08
3167.00	0.00	3169.00	0.00	0.00	0.00
3167.00	0.00	3169.00	0.00	0.00	0.00
3168.00	0.60	3179.00	0.00	0.60	0.03
3177.00	0.00	3179.00	0.00	0.00	0.00
3177.00	0.00	3184.00	0.00	0.00	0.00
3180.00	0.00	3184.00	0.20	0.20	0.01
3182.00	0.00	3188.00	0.70	0.70	0.03
3187.00	0.00	3188.00	0.00	0.00	0.00
3187.00	0.00	3188.00	0.00	0.00	0.00
3195.00	0.00	3196.00	0.00	0.00	0.00
3195.00	0.00	3196.00	0.00	0.00	0.00
3199.00	0.00	3200.00	0.00	0.00	0.00
3199.00	0.00	3200.00	0.00	0.00	0.00
3199.00	0.00	3205.00	0.00	0.00	0.00
3215.00	0.00	3219.00	0.00	0.00	0.00
3216.00	0.00	3219.00	0.00	0.00	0.00
3217.00	0.10	3226.00	0.40	0.50	0.02
3223.00	0.00	3232.00	0.00	0.00	0.00
3225.00	0.20	3234.00	0.00	0.20	0.01

1

Frequency (cm^{-1})	λ_1 (cm^{-1}) ($S_0 \rightarrow S_1$)	Frequency (cm^{-1})	λ_2 (cm^{-1}) ($S_1 \rightarrow S_0$)	λ_{sum} (cm^{-1})	Contribution (%)
23.00	25.50	20.00	19.70	45.20	3.52
24.00	0.00	24.00	0.00	0.00	0.00
36.00	0.00	30.00	0.00	0.00	0.00
45.00	0.00	43.00	0.80	0.80	0.06
54.00	0.40	52.00	2.00	2.40	0.19
54.00	0.00	52.00	0.00	0.00	0.00
61.00	1.30	62.00	0.20	1.50	0.12
68.00	0.00	66.00	0.00	0.00	0.00
73.00	0.00	72.00	0.00	0.00	0.00
81.00	0.10	80.00	0.10	0.20	0.02
116.00	7.20	115.00	5.70	12.90	1.00
118.00	0.00	116.00	0.00	0.00	0.00
151.00	0.00	148.00	0.00	0.00	0.00
162.00	24.90	161.00	28.10	53.00	4.13
200.00	3.80	187.00	0.00	3.80	0.30
209.00	0.00	201.00	4.60	4.60	0.36
216.00	26.70	209.00	0.00	26.70	2.08
220.00	0.00	215.00	29.80	29.80	2.32
227.00	0.00	218.00	2.20	2.20	0.17
249.00	0.00	239.00	0.00	0.00	0.00
250.00	1.70	245.00	1.00	2.70	0.21

264.00	0.00	264.00	0.10	0.10	0.01
281.00	0.00	277.00	0.00	0.00	0.00
309.00	0.00	299.00	0.00	0.00	0.00
330.00	0.00	327.00	0.70	0.70	0.05
330.00	0.00	327.00	0.00	0.00	0.00
384.00	2.00	367.00	0.00	2.00	0.16
393.00	0.00	377.00	2.10	2.10	0.16
424.00	0.00	420.00	0.00	0.00	0.00
424.00	0.00	420.00	0.00	0.00	0.00
438.00	0.00	429.00	0.00	0.00	0.00
449.00	0.00	449.00	0.00	0.00	0.00
462.00	0.00	452.00	0.50	0.50	0.04
466.00	0.20	460.00	0.80	1.00	0.08
486.00	6.00	475.00	0.00	6.00	0.47
488.00	0.00	477.00	5.90	5.90	0.46
490.00	0.00	480.00	0.00	0.00	0.00
499.00	25.30	493.00	17.90	43.20	3.36
508.00	0.00	503.00	0.00	0.00	0.00
529.00	0.00	523.00	3.60	3.60	0.28
533.00	0.00	527.00	0.00	0.00	0.00
547.00	30.80	544.00	0.00	30.80	2.40
551.00	0.00	545.00	33.80	33.80	2.63
599.00	30.60	570.00	0.00	30.60	2.38
600.00	0.00	590.00	33.00	33.00	2.57
610.00	17.50	602.00	0.00	17.50	1.36
612.00	0.00	603.00	20.30	20.30	1.58
631.00	3.90	626.00	2.00	5.90	0.46
639.00	0.00	627.00	0.00	0.00	0.00
647.00	0.00	638.00	0.00	0.00	0.00
649.00	19.90	641.00	0.00	19.90	1.55
656.00	0.00	643.00	12.30	12.30	0.96
660.00	3.90	651.00	6.40	10.30	0.80
668.00	0.10	664.00	1.70	1.80	0.14
683.00	0.00	674.00	0.00	0.00	0.00
699.00	0.00	683.00	0.00	0.00	0.00
702.00	0.00	698.00	0.00	0.00	0.00
707.00	0.00	700.00	0.00	0.00	0.00
716.00	5.50	709.00	0.00	5.50	0.43
718.00	0.00	714.00	14.00	14.00	1.09
720.00	25.60	716.00	0.00	25.60	1.99
747.00	0.00	716.00	17.50	17.50	1.36
758.00	7.50	750.00	5.00	12.50	0.97
764.00	0.00	751.00	2.50	2.50	0.19
774.00	0.00	755.00	0.00	0.00	0.00
785.00	0.00	778.00	0.00	0.00	0.00
798.00	0.00	780.00	0.00	0.00	0.00
805.00	0.70	783.00	0.00	0.70	0.05
805.00	0.00	783.00	0.00	0.00	0.00
810.00	0.00	796.00	0.80	0.80	0.06
818.00	0.10	797.00	0.00	0.10	0.01
820.00	0.00	800.00	0.00	0.00	0.00

822.00	0.60	821.00	0.40	1.00	0.08
855.00	0.60	851.00	0.00	0.60	0.05
857.00	0.00	853.00	0.00	0.00	0.00
864.00	3.70	853.00	0.00	3.70	0.29
867.00	0.00	870.00	1.60	1.60	0.12
877.00	6.40	874.00	7.30	13.70	1.07
884.00	0.00	882.00	0.00	0.00	0.00
884.00	0.00	883.00	0.10	0.10	0.01
910.00	0.20	896.00	0.00	0.20	0.02
936.00	0.00	897.00	0.10	0.10	0.01
951.00	0.00	908.00	1.40	1.40	0.11
959.00	0.00	930.00	0.00	0.00	0.00
959.00	0.00	946.00	0.00	0.00	0.00
974.00	0.00	948.00	0.00	0.00	0.00
982.00	0.00	967.00	0.00	0.00	0.00
986.00	0.00	979.00	1.70	1.70	0.13
986.00	0.00	982.00	0.00	0.00	0.00
992.00	0.60	982.00	0.00	0.60	0.05
1008.00	0.00	1006.00	2.30	2.30	0.18
1008.00	0.30	1006.00	0.00	0.30	0.02
1014.00	9.10	1009.00	9.70	18.80	1.46
1025.00	0.00	1021.00	0.00	0.00	0.00
1026.00	0.30	1023.00	0.00	0.30	0.02
1045.00	0.00	1024.00	0.30	0.30	0.02
1048.00	0.00	1048.00	0.00	0.00	0.00
1050.00	0.10	1050.00	0.10	0.20	0.02
1077.00	0.00	1073.00	0.00	0.00	0.00
1098.00	0.00	1097.00	0.00	0.00	0.00
1098.00	0.00	1097.00	0.00	0.00	0.00
1102.00	1.40	1099.00	0.00	1.40	0.11
1112.00	0.00	1100.00	1.90	1.90	0.15
1125.00	0.10	1122.00	1.30	1.40	0.11
1138.00	3.80	1133.00	6.10	9.90	0.77
1168.00	0.00	1142.00	0.00	0.00	0.00
1183.00	0.00	1176.00	0.00	0.00	0.00
1183.00	0.00	1183.00	0.00	0.00	0.00
1190.00	0.10	1183.00	0.00	0.10	0.01
1192.00	0.00	1190.00	0.00	0.00	0.00
1192.00	0.00	1191.00	0.00	0.00	0.00
1208.00	0.00	1194.00	0.00	0.00	0.00
1232.00	0.00	1207.00	1.90	1.90	0.15
1236.00	0.20	1214.00	0.00	0.20	0.02
1246.00	6.30	1227.00	3.10	9.40	0.73
1251.00	0.00	1240.00	0.00	0.00	0.00
1262.00	0.00	1244.00	0.00	0.00	0.00
1289.00	1.70	1248.00	0.30	2.00	0.16
1303.00	8.90	1264.00	19.00	27.90	2.17
1309.00	0.00	1276.00	0.00	0.00	0.00
1322.00	0.00	1289.00	0.00	0.00	0.00
1322.00	0.10	1296.00	2.20	2.30	0.18
1334.00	42.90	1323.00	0.00	42.90	3.34

1340.00	0.00	1324.00	0.20	0.20	0.02
1344.00	0.00	1339.00	0.00	0.00	0.00
1348.00	0.30	1347.00	0.00	0.30	0.02
1348.00	0.00	1347.00	0.00	0.00	0.00
1359.00	61.50	1348.00	0.00	61.50	4.79
1362.00	0.00	1356.00	140.40	140.40	10.93
1378.00	0.00	1369.00	0.00	0.00	0.00
1383.00	0.00	1379.00	0.90	0.90	0.07
1399.00	0.00	1384.00	32.10	32.10	2.50
1418.00	3.40	1388.00	0.00	3.40	0.26
1458.00	0.00	1416.00	0.00	0.00	0.00
1463.00	52.40	1421.00	26.50	78.90	6.14
1484.00	0.40	1445.00	0.00	0.40	0.03
1484.00	0.00	1478.00	7.80	7.80	0.61
1498.00	0.00	1483.00	0.00	0.00	0.00
1506.00	14.00	1483.00	0.00	14.00	1.09
1529.00	0.00	1492.00	0.00	0.00	0.00
1529.00	3.50	1506.00	6.00	9.50	0.74
1532.00	0.00	1527.00	0.00	0.00	0.00
1534.00	3.70	1527.00	0.20	3.90	0.30
1589.00	0.00	1541.00	38.50	38.50	3.00
1589.00	65.20	1542.00	0.00	65.20	5.08
1606.00	0.00	1560.00	0.00	0.00	0.00
1622.00	42.30	1565.00	0.20	42.50	3.31
1623.00	0.00	1586.00	9.00	9.00	0.70
1625.00	75.80	1599.00	0.00	75.80	5.90
1632.00	0.20	1627.00	0.00	0.20	0.02
1632.00	0.00	1627.00	0.20	0.20	0.02
1646.00	0.50	1637.00	1.20	1.70	0.13
1646.00	0.00	1638.00	0.00	0.00	0.00
3168.00	0.00	3169.00	0.00	0.00	0.00
3168.00	0.00	3169.00	0.00	0.00	0.00
3168.00	0.30	3178.00	0.00	0.30	0.02
3169.00	0.00	3178.00	0.10	0.10	0.01
3169.00	0.70	3178.00	0.00	0.70	0.05
3177.00	0.00	3179.00	0.40	0.40	0.03
3177.00	0.00	3184.00	0.50	0.50	0.04
3187.00	0.00	3188.00	0.00	0.00	0.00
3187.00	0.00	3188.00	0.00	0.00	0.00
3195.00	0.00	3194.00	0.00	0.00	0.00
3195.00	0.00	3194.00	0.00	0.00	0.00
3195.00	0.00	3196.00	0.00	0.00	0.00
3195.00	0.00	3196.00	0.00	0.00	0.00
3199.00	0.00	3200.00	0.00	0.00	0.00
3199.00	0.00	3200.00	0.00	0.00	0.00
3218.00	0.00	3220.00	0.00	0.00	0.00
3220.00	0.00	3220.00	0.10	0.10	0.01
3220.00	0.00	3222.00	0.00	0.00	0.00
3221.00	0.00	3224.00	0.00	0.00	0.00
3240.00	0.00	3235.00	0.00	0.00	0.00
3240.00	0.00	3235.00	0.00	0.00	0.00

2

Frequency (cm^{-1})	λ_1 (cm^{-1}) ($S_0 \rightarrow S_1$)	Frequency (cm^{-1})	λ_2 (cm^{-1}) ($S_1 \rightarrow S_0$)	λ_{sum} (cm^{-1})	Contribution (%)
21.00	22.80	21.00	23.50	46.30	2.21
24.00	0.00	26.00	0.40	0.40	0.02
37.00	0.00	36.00	0.40	0.40	0.02
47.00	0.10	48.00	0.20	0.30	0.01
52.00	0.20	50.00	0.10	0.30	0.01
53.00	0.40	52.00	0.20	0.60	0.03
63.00	0.00	63.00	0.00	0.00	0.00
71.00	0.00	68.00	6.00	6.00	0.29
74.00	0.00	72.00	0.30	0.30	0.01
78.00	16.70	78.00	7.90	24.60	1.17
107.00	12.40	105.00	9.50	21.90	1.04
123.00	0.00	118.00	0.20	0.20	0.01
148.00	0.00	149.00	1.10	1.10	0.05
196.00	44.50	187.00	31.30	75.80	3.61
202.00	0.90	199.00	6.20	7.10	0.34
206.00	0.00	203.00	4.80	4.80	0.23
218.00	0.10	217.00	0.20	0.30	0.01
228.00	0.00	223.00	0.10	0.10	0.00
237.00	0.00	233.00	0.20	0.20	0.01
249.00	0.00	240.00	0.00	0.00	0.00
260.00	4.00	255.00	15.90	19.90	0.95
268.00	26.30	263.00	22.30	48.60	2.32
278.00	0.00	276.00	0.20	0.20	0.01
318.00	0.00	316.00	0.10	0.10	0.00
331.00	0.00	324.00	1.90	1.90	0.09
349.00	9.30	337.00	0.30	9.60	0.46
377.00	23.80	374.00	9.30	33.10	1.58
395.00	0.00	378.00	16.40	16.40	0.78
424.00	0.00	406.00	0.70	0.70	0.03
424.00	0.00	421.00	0.00	0.00	0.00
439.00	0.00	422.00	0.00	0.00	0.00
443.00	0.40	438.00	0.50	0.90	0.04
454.00	13.90	451.00	15.30	29.20	1.39
459.00	0.00	455.00	0.90	0.90	0.04
469.00	16.60	466.00	0.50	17.10	0.82
473.00	0.00	471.00	1.40	1.40	0.07
495.00	0.00	481.00	15.00	15.00	0.72
506.00	46.10	498.00	24.50	70.60	3.37
511.00	0.00	501.00	7.10	7.10	0.34
527.00	0.00	527.00	1.00	1.00	0.05
543.00	2.50	529.00	1.20	3.70	0.18
551.00	11.90	541.00	4.80	16.70	0.80
559.00	0.00	552.00	0.20	0.20	0.01

615.00	3.20	563.00	0.40	3.60	0.17
620.00	0.00	601.00	0.50	0.50	0.02
621.00	11.40	611.00	0.30	11.70	0.56
630.00	0.00	616.00	10.70	10.70	0.51
633.00	0.70	625.00	6.10	6.80	0.32
639.00	0.00	630.00	1.80	1.80	0.09
641.00	11.20	632.00	0.00	11.20	0.53
643.00	0.00	635.00	11.30	11.30	0.54
646.00	7.50	639.00	0.00	7.50	0.36
652.00	0.00	653.00	0.20	0.20	0.01
663.00	0.00	661.00	0.00	0.00	0.00
668.00	1.70	666.00	0.40	2.10	0.10
673.00	0.00	684.00	2.40	2.40	0.11
689.00	0.50	696.00	1.30	1.80	0.09
717.00	0.00	710.00	2.20	2.20	0.10
717.00	0.20	714.00	1.30	1.50	0.07
724.00	8.60	714.00	5.10	13.70	0.65
741.00	0.00	719.00	2.70	2.70	0.13
749.00	0.00	728.00	0.60	0.60	0.03
759.00	0.10	735.00	12.90	13.00	0.62
768.00	0.00	746.00	0.90	0.90	0.04
773.00	0.90	761.00	0.20	1.10	0.05
781.00	0.20	774.00	0.50	0.70	0.03
783.00	0.00	776.00	0.10	0.10	0.00
805.00	0.00	785.00	0.10	0.10	0.00
807.00	0.00	799.00	0.10	0.10	0.00
815.00	7.20	801.00	0.20	7.40	0.35
817.00	0.00	814.00	23.90	23.90	1.14
828.00	0.00	822.00	2.20	2.20	0.10
844.00	1.80	831.00	19.60	21.40	1.02
855.00	0.30	834.00	0.00	0.30	0.01
857.00	0.00	835.00	20.70	20.70	0.99
864.00	2.90	840.00	0.30	3.20	0.15
865.00	0.00	848.00	7.80	7.80	0.37
872.00	7.80	850.00	0.10	7.90	0.38
873.00	0.00	856.00	0.00	0.00	0.00
882.00	0.10	874.00	2.50	2.60	0.12
891.00	0.90	880.00	4.50	5.40	0.26
896.00	0.00	880.00	2.90	2.90	0.14
912.00	0.00	891.00	0.70	0.70	0.03
942.00	0.00	913.00	0.40	0.40	0.02
952.00	0.10	938.00	0.10	0.20	0.01
971.00	0.00	951.00	0.00	0.00	0.00
972.00	0.10	972.00	0.10	0.20	0.01
972.00	0.00	973.00	0.10	0.10	0.00
986.00	0.00	977.00	0.00	0.00	0.00
986.00	0.00	987.00	0.00	0.00	0.00
1002.00	0.00	993.00	0.10	0.10	0.00
1008.00	0.30	1002.00	0.20	0.50	0.02
1008.00	0.00	1007.00	0.40	0.40	0.02
1013.00	1.20	1012.00	0.10	1.30	0.06

1025.00	0.00	1024.00	3.60	3.60	0.17
1026.00	0.50	1025.00	0.00	0.50	0.02
1047.00	0.70	1037.00	1.30	2.00	0.10
1047.00	0.00	1048.00	3.50	3.50	0.17
1060.00	1.50	1052.00	0.60	2.10	0.10
1070.00	0.00	1071.00	11.40	11.40	0.54
1098.00	0.00	1096.00	0.00	0.00	0.00
1098.00	0.00	1100.00	0.10	0.10	0.00
1128.00	0.10	1123.00	0.10	0.20	0.01
1146.00	0.00	1127.00	0.10	0.10	0.00
1171.00	3.80	1162.00	0.60	4.40	0.21
1172.00	0.00	1172.00	2.10	2.10	0.10
1183.00	0.10	1181.00	0.90	1.00	0.05
1183.00	0.00	1182.00	0.10	0.10	0.00
1192.00	0.20	1185.00	0.10	0.30	0.01
1193.00	0.00	1190.00	0.00	0.00	0.00
1198.00	3.20	1193.00	0.00	3.20	0.15
1208.00	0.00	1194.00	0.20	0.20	0.01
1217.00	0.00	1205.00	1.10	1.10	0.05
1220.00	2.30	1213.00	0.10	2.40	0.11
1223.00	0.00	1218.00	0.10	0.10	0.00
1225.00	1.70	1225.00	0.20	1.90	0.09
1250.00	0.00	1239.00	5.20	5.20	0.25
1265.00	0.00	1248.00	0.50	0.50	0.02
1303.00	4.00	1273.00	6.20	10.20	0.49
1304.00	0.00	1289.00	1.50	1.50	0.07
1319.00	0.00	1314.00	4.40	4.40	0.21
1319.00	20.00	1324.00	0.00	20.00	0.95
1322.00	0.20	1324.00	0.60	0.80	0.04
1322.00	0.00	1329.00	30.50	30.50	1.45
1348.00	0.20	1346.00	0.00	0.20	0.01
1348.00	0.00	1349.00	0.00	0.00	0.00
1356.00	0.00	1352.00	9.00	9.00	0.43
1377.00	0.00	1367.00	14.70	14.70	0.70
1387.00	160.90	1373.00	30.60	191.50	9.13
1397.00	0.00	1380.00	31.10	31.10	1.48
1400.00	0.70	1388.00	55.20	55.90	2.67
1411.00	4.50	1404.00	12.30	16.80	0.80
1413.00	0.00	1415.00	51.50	51.50	2.46
1441.00	0.00	1422.00	21.60	21.60	1.03
1443.00	207.30	1440.00	101.50	308.80	14.73
1484.00	0.00	1466.00	11.80	11.80	0.56
1484.00	0.00	1482.00	0.00	0.00	0.00
1487.00	0.00	1485.00	0.00	0.00	0.00
1491.00	15.60	1488.00	17.20	32.80	1.56
1512.00	0.00	1493.00	21.00	21.00	1.00
1522.00	78.70	1509.00	9.80	88.50	4.22
1530.00	0.00	1528.00	7.00	7.00	0.33
1532.00	35.80	1528.00	0.20	36.00	1.72
1569.00	62.70	1544.00	19.30	82.00	3.91
1569.00	0.00	1545.00	114.80	114.80	5.47

1610.00	0.00	1565.00	41.70	41.70	1.99
1624.00	49.90	1579.00	0.30	50.20	2.39
1631.00	1.10	1610.00	0.20	1.30	0.06
1632.00	0.00	1624.00	23.00	23.00	1.10
1639.00	0.00	1627.00	2.40	2.40	0.11
1641.00	20.00	1632.00	0.10	20.10	0.96
1647.00	15.60	1639.00	0.50	16.10	0.77
1648.00	0.00	1643.00	2.50	2.50	0.12
3168.00	0.00	3166.00	0.00	0.00	0.00
3168.00	0.00	3170.00	0.50	0.50	0.02
3169.00	0.10	3172.00	0.00	0.10	0.00
3172.00	0.00	3176.00	0.10	0.10	0.00
3172.00	0.40	3176.00	0.10	0.50	0.02
3177.00	0.00	3181.00	0.20	0.20	0.01
3177.00	0.00	3184.00	2.10	2.10	0.10
3187.00	0.00	3186.00	0.00	0.00	0.00
3187.00	0.00	3190.00	0.00	0.00	0.00
3195.00	0.00	3195.00	0.00	0.00	0.00
3195.00	0.00	3197.00	0.20	0.20	0.01
3199.00	0.00	3199.00	0.80	0.80	0.04
3199.00	0.00	3201.00	1.00	1.00	0.05
3219.00	0.00	3220.00	0.00	0.00	0.00
3220.00	0.20	3221.00	0.10	0.30	0.01
3220.00	0.00	3225.00	0.30	0.30	0.01
3222.00	0.10	3227.00	0.10	0.20	0.01
3245.00	0.00	3242.00	4.60	4.60	0.22
3245.00	0.00	3245.00	1.90	1.90	0.09
3277.00	0.10	3251.00	12.10	12.20	0.58
3278.00	0.00	3282.00	3.50	3.50	0.17

3

Frequency (cm^{-1})	λ_1 (cm^{-1}) ($S_0 \rightarrow S_1$)	Frequency (cm^{-1})	λ_2 (cm^{-1}) ($S_1 \rightarrow S_0$)	λ_{sum} (cm^{-1})	Contribution (%)
21.00	38.40	19.00	28.20	66.60	3.72
25.00	0.00	24.00	0.00	0.00	0.00
40.00	0.00	33.00	0.00	0.00	0.00
46.00	0.10	45.00	3.40	3.50	0.20
53.00	0.20	52.00	9.90	10.10	0.56
55.00	10.40	57.00	2.20	12.60	0.70
63.00	0.00	58.00	0.00	0.00	0.00
69.00	0.00	65.00	0.00	0.00	0.00
76.00	0.00	75.00	0.00	0.00	0.00
79.00	14.20	77.00	11.60	25.80	1.44
116.00	0.20	116.00	0.00	0.20	0.01
125.00	0.00	116.00	0.00	0.00	0.00
146.00	0.00	149.00	0.00	0.00	0.00
193.00	43.80	186.00	46.80	90.60	5.06

203.00	1.80	192.00	0.00	1.80	0.10
211.00	0.00	206.00	4.20	4.20	0.23
218.00	8.10	216.00	8.80	16.90	0.94
225.00	0.00	217.00	0.00	0.00	0.00
236.00	0.00	225.00	2.30	2.30	0.13
255.00	0.00	238.00	0.00	0.00	0.00
259.00	0.70	257.00	0.50	1.20	0.07
272.00	3.80	266.00	0.00	3.80	0.21
273.00	0.00	268.00	3.40	3.40	0.19
308.00	0.00	305.00	0.00	0.00	0.00
324.00	0.00	321.00	0.00	0.00	0.00
335.00	2.10	331.00	3.80	5.90	0.33
350.00	6.30	343.00	7.30	13.60	0.76
398.00	0.00	371.00	0.00	0.00	0.00
424.00	0.00	419.00	0.00	0.00	0.00
424.00	0.00	420.00	0.00	0.00	0.00
441.00	0.00	432.00	0.00	0.00	0.00
447.00	3.00	441.00	0.00	3.00	0.17
447.00	0.00	448.00	10.40	10.40	0.58
458.00	0.40	450.00	0.90	1.30	0.07
471.00	0.00	465.00	0.00	0.00	0.00
484.00	14.50	482.00	4.60	19.10	1.07
499.00	0.00	491.00	0.00	0.00	0.00
512.00	8.50	503.00	0.00	8.50	0.48
513.00	0.00	513.00	7.50	7.50	0.42
521.00	0.00	525.00	0.00	0.00	0.00
530.00	0.00	528.00	0.20	0.20	0.01
559.00	0.00	552.00	0.00	0.00	0.00
561.00	52.40	555.00	64.30	116.70	6.52
579.00	16.60	569.00	11.30	27.90	1.56
589.00	0.00	578.00	0.00	0.00	0.00
621.00	2.60	591.00	0.00	2.60	0.15
624.00	0.00	604.00	0.60	0.60	0.03
633.00	0.80	622.00	0.00	0.80	0.04
640.00	0.00	629.00	6.60	6.60	0.37
646.00	0.00	635.00	0.00	0.00	0.00
646.00	38.30	638.00	0.00	38.30	2.14
654.00	0.00	638.00	25.50	25.50	1.43
661.00	0.30	652.00	0.00	0.30	0.02
668.00	0.10	664.00	0.70	0.80	0.04
690.00	0.00	671.00	0.00	0.00	0.00
712.00	0.80	685.00	0.00	0.80	0.04
715.00	0.00	690.00	0.30	0.30	0.02
716.00	0.30	703.00	0.00	0.30	0.02
718.00	0.00	711.00	0.90	0.90	0.05
723.00	8.00	715.00	0.00	8.00	0.45
731.00	0.00	718.00	7.00	7.00	0.39
751.00	0.00	724.00	0.00	0.00	0.00
752.00	0.00	740.00	0.00	0.00	0.00
774.00	3.20	751.00	1.30	4.50	0.25
780.00	0.40	752.00	0.00	0.40	0.02

784.00	0.00	768.00	1.00	1.00	0.06
801.00	25.30	768.00	0.00	25.30	1.41
803.00	0.00	773.00	1.60	1.60	0.09
805.00	0.00	789.00	0.00	0.00	0.00
810.00	0.20	792.00	25.80	26.00	1.45
814.00	0.00	797.00	0.00	0.00	0.00
825.00	0.00	811.00	0.00	0.00	0.00
830.00	11.10	822.00	9.50	20.60	1.15
855.00	0.80	852.00	0.00	0.80	0.04
857.00	0.00	853.00	0.00	0.00	0.00
862.00	3.70	872.00	2.10	5.80	0.32
879.00	4.50	873.00	7.10	11.60	0.65
883.00	0.00	875.00	0.00	0.00	0.00
908.00	0.00	881.00	0.00	0.00	0.00
913.00	0.10	883.00	0.20	0.30	0.02
918.00	0.00	900.00	0.00	0.00	0.00
941.00	0.00	909.00	0.00	0.00	0.00
949.00	9.50	913.00	0.60	10.10	0.56
952.00	0.00	937.00	0.00	0.00	0.00
953.00	0.00	939.00	11.10	11.10	0.62
955.00	0.40	939.00	0.00	0.40	0.02
972.00	0.00	950.00	1.00	1.00	0.06
974.00	0.00	954.00	0.00	0.00	0.00
986.00	0.00	982.00	0.00	0.00	0.00
986.00	0.00	982.00	0.00	0.00	0.00
1008.00	0.00	995.00	0.00	0.00	0.00
1008.00	0.10	1007.00	0.10	0.20	0.01
1025.00	0.00	1007.00	0.00	0.00	0.00
1025.00	0.20	1022.00	1.30	1.50	0.08
1037.00	4.90	1023.00	0.00	4.90	0.27
1041.00	0.00	1026.00	6.90	6.90	0.39
1048.00	0.00	1046.00	0.00	0.00	0.00
1052.00	0.40	1051.00	0.00	0.40	0.02
1082.00	0.00	1081.00	0.00	0.00	0.00
1098.00	0.00	1097.00	0.00	0.00	0.00
1098.00	0.00	1097.00	0.00	0.00	0.00
1112.00	1.40	1101.00	0.00	1.40	0.08
1115.00	0.00	1107.00	2.40	2.40	0.13
1126.00	3.60	1115.00	0.00	3.60	0.20
1153.00	10.40	1123.00	10.30	20.70	1.16
1183.00	0.00	1151.00	17.40	17.40	0.97
1183.00	0.00	1183.00	0.20	0.20	0.01
1192.00	0.00	1183.00	0.00	0.00	0.00
1192.00	0.00	1184.00	2.90	2.90	0.16
1201.00	0.00	1191.00	0.00	0.00	0.00
1203.00	3.70	1191.00	0.90	4.60	0.26
1209.00	0.00	1194.00	0.00	0.00	0.00
1221.00	0.00	1197.00	6.60	6.60	0.37
1232.00	9.30	1199.00	0.00	9.30	0.52
1243.00	0.50	1217.00	0.00	0.50	0.03
1248.00	0.00	1233.00	3.50	3.50	0.20

1261.00	0.00	1238.00	1.60	1.60	0.09
1282.00	0.30	1253.00	0.00	0.30	0.02
1299.00	19.70	1269.00	17.40	37.10	2.07
1309.00	0.00	1274.00	0.00	0.00	0.00
1321.00	0.00	1306.00	0.00	0.00	0.00
1322.00	0.00	1313.00	21.30	21.30	1.19
1322.00	0.00	1324.00	0.00	0.00	0.00
1332.00	1.60	1324.00	0.60	2.20	0.12
1347.00	0.00	1325.00	0.00	0.00	0.00
1347.00	27.70	1332.00	16.90	44.60	2.49
1348.00	0.00	1347.00	0.00	0.00	0.00
1348.00	69.10	1347.00	0.00	69.10	3.86
1364.00	0.00	1349.00	0.00	0.00	0.00
1387.00	0.00	1368.00	184.40	184.40	10.31
1392.00	38.30	1373.00	0.00	38.30	2.14
1423.00	0.00	1404.00	0.00	0.00	0.00
1431.00	3.80	1406.00	18.40	22.20	1.24
1453.00	0.00	1408.00	0.00	0.00	0.00
1462.00	86.90	1426.00	60.40	147.30	8.23
1484.00	1.10	1453.00	0.00	1.10	0.06
1484.00	0.00	1483.00	0.00	0.00	0.00
1491.00	0.00	1483.00	0.30	0.30	0.02
1493.00	25.70	1501.00	18.00	43.70	2.44
1530.00	0.00	1513.00	37.60	37.60	2.10
1530.00	0.40	1516.00	0.00	0.40	0.02
1536.00	0.00	1521.00	0.00	0.00	0.00
1543.00	17.30	1526.00	0.00	17.30	0.97
1581.00	0.00	1527.00	0.10	0.10	0.01
1583.00	50.60	1535.00	21.60	72.20	4.04
1603.00	0.00	1544.00	0.00	0.00	0.00
1613.00	104.80	1552.00	8.20	113.00	6.32
1618.00	0.00	1581.00	6.10	6.10	0.34
1625.00	165.20	1596.00	0.00	165.20	9.23
1631.00	0.30	1626.00	0.00	0.30	0.02
1631.00	0.00	1627.00	0.20	0.20	0.01
1645.00	0.20	1636.00	2.90	3.10	0.17
1646.00	0.00	1636.00	0.00	0.00	0.00
3168.00	0.00	3170.00	0.00	0.00	0.00
3168.00	0.00	3170.00	0.00	0.00	0.00
3168.00	0.40	3179.00	0.00	0.40	0.02
3177.00	0.00	3179.00	0.00	0.00	0.00
3177.00	0.00	3186.00	0.40	0.40	0.02
3179.00	0.00	3188.00	0.00	0.00	0.00
3179.00	0.70	3188.00	0.00	0.70	0.04
3187.00	0.00	3189.00	0.00	0.00	0.00
3187.00	0.00	3189.00	0.50	0.50	0.03
3195.00	0.00	3196.00	0.00	0.00	0.00
3195.00	0.00	3196.00	0.00	0.00	0.00
3199.00	0.00	3200.00	0.00	0.00	0.00
3199.00	0.00	3200.00	0.00	0.00	0.00
3219.00	0.00	3223.00	0.00	0.00	0.00

3222.00	0.00	3224.00	0.10	0.10	0.01
3222.00	0.00	3225.00	0.00	0.00	0.00
3222.00	0.20	3225.00	0.10	0.30	0.02
3227.00	0.00	3227.00	0.00	0.00	0.00
3228.00	0.20	3230.00	0.00	0.20	0.01
3246.00	0.00	3248.00	0.00	0.00	0.00
3246.00	0.00	3248.00	0.10	0.10	0.01

Table S6. Mode-specific reorganization-energy contributions

Material	λ_{total} (cm^{-1})	λ_{total} (eV)	Freq1	Freq2	λ_{sum}	Contribution(%)
DABNA-1	2135.0	0.265	1626.0	1566.0	311.1	14.571
1	1284.0	0.159	1362.0	1356.0	140.4	10.935
2	2097.0	0.260	1443.0	1440.0	308.8	14.726
3	1789.0	0.222	1387.0	1368.0	184.4	10.307

Table S7. The cartesian coordinates of **1**, **2**, and **3** in the S_1 state and T_1 state.

1

	S_1			T_1		
C	0.0000000	1.1805520	0.0000000	0.0000020	1.1726590	-0.0000050
C	1.2042320	1.8892590	-0.0907410	1.2049490	1.8917780	-0.0782190
C	1.2058360	3.3075010	-0.1132310	1.2070410	3.2985110	-0.0961800
C	0.0000000	3.9894600	-0.0000020	0.0000070	3.9863300	-0.0000030
C	-1.2042320	1.8892590	0.0907410	-1.2049420	1.8917820	0.0782110
C	-1.2058350	3.3075010	0.1132290	-1.2070300	3.2985140	0.0961730
N	2.4034760	1.1815630	-0.1554750	2.4138120	1.1752640	-0.1480780
C	2.5244800	-0.1961100	0.1412030	2.5323990	-0.1937480	0.1545100
C	1.3427580	-0.9744040	0.3532170	1.3414330	-0.9743070	0.3520380
B	0.0000000	-0.3477220	0.0000000	0.0000000	-0.3450810	-0.0000040
C	-1.3427590	-0.9744040	-0.3532170	-1.3414350	-0.9743030	-0.3520430
C	-2.5244800	-0.1961100	-0.1412040	-2.5323990	-0.1937410	-0.1545110
N	-2.4034760	1.1815640	0.1554750	-2.4138070	1.1752710	0.1480740
C	3.8096220	-0.7670650	0.2361460	3.8156720	-0.7682330	0.2660300
C	3.9769400	-2.0780590	0.6644710	3.9791440	-2.0746750	0.6973870
C	2.8566070	-2.8213800	1.0740800	2.8484700	-2.8150070	1.1019360
C	1.5729640	-2.2430800	0.9319880	1.5692070	-2.2377340	0.9445630
C	-1.5729640	-2.2430800	-0.9319890	-1.5692150	-2.2377300	-0.9445660
C	-2.8566080	-2.8213790	-1.0740810	-2.8484810	-2.8150010	-1.1019310
C	-3.9769410	-2.0780580	-0.6644730	-3.9791500	-2.0746670	-0.6973750
C	-3.8096220	-0.7670640	-0.2361490	-3.8156740	-0.7682240	-0.2660220
C	2.8054510	-4.1066740	1.7029740	2.7927610	-4.0959230	1.7365360
C	1.5457260	-4.4566520	2.0810170	1.5283720	-4.4451460	2.1029820
S	0.3441630	-3.2639940	1.6694860	0.3338960	-3.2570080	1.6720100
S	-0.3441630	-3.2639950	-1.6694850	-0.3339100	-3.2570080	-1.6720170
C	-1.5457270	-4.4566520	-2.0810180	-1.5283920	-4.4451440	-2.1029810
C	-2.8054520	-4.1066730	-1.7029750	-2.7927780	-4.0959180	-1.7365290

C	3.6124020	1.9296250	-0.3759190	3.6187510	1.9251370	-0.3786610
C	4.3296030	2.4280760	0.7085670	4.3361460	2.4411100	0.6976320
C	5.4883390	3.1658320	0.4841330	5.4952190	3.1743620	0.4620330
C	5.9273470	3.3975770	-0.8189740	5.9336540	3.3875920	-0.8439980
C	5.2077520	2.8905780	-1.8982140	5.2119150	2.8665320	-1.9154210
C	4.0469680	2.1515930	-1.6782690	4.0512480	2.1321380	-1.6841540
C	-3.6124010	1.9296260	0.3759200	-3.6187440	1.9251480	0.3786600
C	-4.0469690	2.1515900	1.6782700	-4.0512350	2.1321510	1.6841540
C	-4.3296010	2.4280790	-0.7085660	-4.3361400	2.4411240	-0.6976300
C	-5.4883370	3.1658350	-0.4841310	-5.4952090	3.1743790	-0.4620280
C	-5.9273470	3.3975780	0.8189760	-5.9336400	3.3876100	0.8440050
C	-5.2077530	2.8905760	1.8982150	-5.2118990	2.8665480	1.9154250
H	2.1301260	3.8574830	-0.2084080	2.1328770	3.8485720	-0.1803960
H	0.0000010	5.0723820	-0.0000030	0.0000080	5.0688560	-0.0000030
H	-2.1301250	3.8574830	0.2084050	-2.1328630	3.8485790	0.1803910
H	4.6804440	-0.1798870	-0.0132090	4.6887650	-0.1797310	0.0271620
H	4.9704420	-2.5046640	0.7308520	4.9703120	-2.5037740	0.7760770
H	-4.9704420	-2.5046620	-0.7308550	-4.9703200	-2.5037630	-0.7760590
H	-4.6804450	-0.1798850	0.0132070	-4.6887640	-0.1797190	-0.0271490
H	3.6775150	-4.7207480	1.8861370	3.6634780	-4.7078970	1.9320300
H	1.2397390	-5.3555550	2.5948100	1.2176260	-5.3416930	2.6180600
H	-1.2397410	-5.3555550	-2.5948100	-1.2176500	-5.3416930	-2.6180590
H	-3.6775160	-4.7207470	-1.8861380	-3.6634970	-4.7078900	-1.9320170
H	3.9730490	2.2329860	1.7123520	3.9802300	2.2613730	1.7044830
H	6.0470950	3.5597390	1.3247210	6.0552810	3.5787990	1.2967740
H	6.8295980	3.9722420	-0.9917680	6.8364900	3.9585870	-1.0255540
H	5.5481670	3.0686250	-2.9113010	5.5512440	3.0305320	-2.9312530
H	3.4731970	1.7460860	-2.5022980	3.4758530	1.7169960	-2.5022200
H	-3.4731980	1.7460810	2.5022980	-3.4758400	1.7170060	2.5022190
H	-3.9730460	2.2329920	-1.7123510	-3.9802270	2.2613850	-1.7044830
H	-6.0470920	3.5597460	-1.3247180	-6.0552730	3.5788190	-1.2967670
H	-6.8295970	3.9722420	0.9917710	-6.8364730	3.9586080	1.0255630
H	-5.5481690	3.0686200	2.9113020	-5.5512240	3.0305480	2.9312580

2

	S_1			T_1		
C	-0.0351930	1.2746930	-0.0550690	0.0417540	1.2735950	0.0019450
C	1.1569180	2.0246180	-0.1151540	1.2776510	1.9621780	-0.0584970
C	1.0974560	3.4416660	-0.1458360	1.3070340	3.3586790	-0.0536510
C	-0.1277770	4.0718550	-0.0737400	0.1065600	4.0648130	0.0423760
C	-1.2615150	1.9562310	0.0255860	-1.1509760	2.0200840	0.0745920
C	-1.3224540	3.3521880	0.0282200	-1.1186860	3.4286650	0.1151000
N	2.3662040	1.3638780	-0.1341490	2.4684430	1.2239190	-0.1476600
C	2.5213410	-0.0288800	0.1014850	2.5665110	-0.1538390	0.0823600
C	1.3637720	-0.8356120	0.3840110	1.3409110	-0.9205370	0.3113880
B	0.0371020	-0.2461020	0.0577030	0.0022360	-0.2433580	-0.0105780
C	-1.3473040	-0.9216070	-0.2410180	-1.3687130	-0.8455230	-0.3309350
C	-2.5352260	-0.1552420	-0.0779300	-2.4948730	-0.0287380	-0.1493210
N	-2.4637680	1.2049870	0.1297970	-2.3711320	1.3380520	0.1144630
C	3.8025880	-0.5903810	0.0104810	3.8068300	-0.7788060	0.0934900
C	4.0116090	-1.9497900	0.2643560	3.9364630	-2.1359540	0.4553350
C	2.9583510	-2.7694740	0.7300100	2.8136870	-2.8595730	0.8923040
C	1.6319460	-2.1817490	0.8756210	1.5163530	-2.2182480	0.8579600

C	-1.5243920	-2.2210120	-0.7723850	-1.6201700	-2.1807950	-0.8384130
C	-2.8307470	-2.8265440	-0.9192370	-2.9670770	-2.7044770	-0.9359940
C	-3.9796530	-2.0712820	-0.5409410	-4.0581330	-1.8630780	-0.5683340
C	-3.8326910	-0.7760260	-0.1484260	-3.8319320	-0.5716730	-0.2143320
C	2.9928580	-4.0945050	1.1472040	2.7414260	-4.1629650	1.3776600
C	-2.7692970	-4.1041190	-1.4250090	-3.0135570	-3.9923270	-1.4338660
C	3.5579390	2.1469310	-0.3098580	3.6875870	1.9538980	-0.3586360
C	4.2769830	2.5697610	0.8043670	4.4314070	2.3948170	0.7327280
C	5.4214920	3.3427730	0.6315870	5.6075190	3.1083630	0.5209890
C	5.8467140	3.6841870	-0.6521970	6.0373100	3.3763940	-0.7778100
C	5.1273470	3.2509100	-1.7629780	5.2901720	2.9298540	-1.8654550
C	3.9808600	2.4766210	-1.5933490	4.1127390	2.2154150	-1.6568280
C	-3.6850840	1.9389290	0.3485910	-3.5579450	2.1188610	0.3467630
C	-4.1100540	2.1773340	1.6508400	-3.9781120	2.3528530	1.6518910
C	-4.4162960	2.4072200	-0.7390770	-4.2647530	2.6457690	-0.7307030
C	-5.5914440	3.1196460	-0.5175470	-5.4061850	3.4076420	-0.4981430
C	-6.0269310	3.3607790	0.7843060	-5.8347470	3.6427410	0.8071180
C	-5.2866990	2.8904570	1.8668400	-5.1202980	3.1158450	1.8809370
H	2.0030910	4.0264070	-0.2083130	2.2412730	3.8962580	-0.1139780
H	-0.1669610	5.1547480	-0.0844880	0.1386700	5.1483210	0.0579070
H	-2.2616840	3.8769700	0.1107040	-2.0274400	4.0065400	0.1855520
H	4.6458110	0.0237550	-0.2655010	4.6983760	-0.2184700	-0.1443160
H	5.0093400	-2.3614900	0.1685260	4.9172520	-2.5950060	0.4618340
H	-4.9648770	-2.5189470	-0.5898550	-5.0706590	-2.2492750	-0.5974600
H	-4.7072190	-0.1951880	0.1023620	-4.6702710	0.0628570	0.0298990
H	3.8409840	-4.7593030	1.1743080	3.5434770	-4.8782320	1.4698660
H	-3.5821240	-4.7856930	-1.6213610	-3.8804860	-4.6070700	-1.6165920
H	3.9339830	2.2847390	1.7910440	4.0814060	2.1736020	1.7333630
H	5.9814210	3.6772600	1.4968860	6.1875310	3.4548790	1.3681530
H	6.7387920	4.2850210	-0.7851140	6.9532150	3.9320090	-0.9412470
H	5.4589680	3.5110480	-2.7613190	5.6231700	3.1363660	-2.8757060
H	3.4100950	2.1239830	-2.4433770	3.5172280	1.8588430	-2.4880870
H	-3.5173260	1.8071180	2.4780490	-3.4076040	1.9361220	2.4726940
H	-4.0586540	2.2122000	-1.7424960	-3.9145820	2.4524080	-1.7371310
H	-6.1645540	3.4873720	-1.3601150	-5.9594870	3.8177630	-1.3345820
H	-6.9413280	3.9163180	0.9545980	-6.7233740	4.2361590	0.9867220
H	-5.6223000	3.0797270	2.8792710	-5.4510890	3.2983790	2.8964160
S	-1.1321090	-4.5748220	-1.7639610	-1.4384810	-4.5599840	-1.8178390
S	1.4654040	-4.6045680	1.8209190	1.1052600	-4.6112660	1.8873440
C	-0.5133620	-3.0804810	-1.2203020	-0.6967590	-3.0932610	-1.3123410
C	0.7568340	-3.0428200	1.4913300	0.5250940	-3.0379750	1.4049770
H	0.5448910	-2.8878790	-1.2371660	0.3711840	-2.9867420	-1.3840550
H	-0.2689600	-2.8631230	1.7652950	-0.5131590	-2.7888870	1.5440140

3

	S_1			T_1		
C	0.0000010	1.2480550	0.0000000	0.0000010	1.2413800	-0.0000020
C	1.1996090	1.9613990	-0.1084690	1.2009210	1.9653660	-0.0960890
C	1.2003450	3.3866860	-0.1319510	1.2026870	3.3755620	-0.1136670
C	0.0000020	4.0696090	0.0000030	0.0000030	4.0642620	-0.0000040
C	-1.1996070	1.9614000	0.1084690	-1.2009180	1.9653680	0.0960850
C	-1.2003420	3.3866870	0.1319550	-1.2026820	3.3755640	0.1136600
N	2.3963350	1.2719030	-0.1900250	2.4088010	1.2621120	-0.1831570

C	2.5300540	-0.1180020	0.0733170	2.5382950	-0.1152830	0.0853570
C	1.3626250	-0.9129060	0.2952790	1.3604700	-0.9107690	0.2967090
B	0.0000000	-0.2910040	-0.0000020	0.0000000	-0.2836830	-0.0000020
C	-1.3626260	-0.9129050	-0.2952820	-1.3604710	-0.9107670	-0.2967120
C	-2.5300540	-0.1180000	-0.0733180	-2.5382950	-0.1152790	-0.0853590
N	-2.3963340	1.2719050	0.1900250	-2.4087990	1.2621150	0.1831540
C	3.8237100	-0.6664190	0.1104130	3.8300890	-0.6733520	0.1367260
C	4.0269640	-1.9975750	0.4703000	4.0261700	-1.9983360	0.5054830
C	2.9205770	-2.7506210	0.8571940	2.9083210	-2.7447320	0.8901960
C	1.6070030	-2.2242100	0.8129600	1.5987520	-2.2160360	0.8274860
C	-1.6070060	-2.2242090	-0.8129620	-1.5987550	-2.2160340	-0.8274880
C	-2.9205800	-2.7506190	-0.8571920	-2.9083250	-2.7447290	-0.8901960
C	-4.0269650	-1.9975730	-0.4702960	-4.0261730	-1.9983310	-0.5054810
C	-3.8237100	-0.6664160	-0.1104110	-3.8300900	-0.6733470	-0.1367260
C	3.5997990	2.0297100	-0.4126990	3.6076620	2.0216520	-0.4190300
C	4.3260460	2.5130030	0.6723090	4.3384700	2.5203440	0.6561000
C	5.4787250	3.2603330	0.4473060	5.4909170	3.2628480	0.4169030
C	5.9025980	3.5148650	-0.8565550	5.9092510	3.5016340	-0.8913030
C	5.1743980	3.0213650	-1.9362710	5.1741280	2.9969880	-1.9614800
C	4.0194630	2.2730270	-1.7159340	4.0199550	2.2534690	-1.7268000
C	-3.5997970	2.0297130	0.4126980	-3.6076590	2.0216570	0.4190300
C	-4.0194620	2.2730300	1.7159330	-4.0199490	2.2534730	1.7268010
C	-4.3260420	2.5130090	-0.6723100	-4.3384680	2.5203500	-0.6560980
C	-5.4787200	3.2603410	-0.4473070	-5.4909140	3.2628560	-0.4168980
C	-5.9025930	3.5148730	0.8565530	-5.9092440	3.5016410	0.8913090
C	-5.1743950	3.0213690	1.9362700	-5.1741200	2.9969940	1.9614840
H	2.1241130	3.9338480	-0.2420630	2.1278990	3.9238930	-0.2107870
H	0.0000030	5.1524650	0.0000050	0.0000040	5.1467600	-0.0000050
H	-2.1241090	3.9338490	0.2420690	-2.1278930	3.9238960	0.2107800
H	4.6801670	-0.0578630	-0.1351870	4.6901150	-0.0671330	-0.1032340
H	5.0266980	-2.4116740	0.4868770	5.0230550	-2.4179260	0.5350090
H	-5.0267000	-2.4116710	-0.4868710	-5.0230590	-2.4179190	-0.5350060
H	-4.6801660	-0.0578590	0.1351900	-4.6901140	-0.0671270	0.1032350
H	3.9819240	2.2988710	1.6765320	3.9985420	2.3201560	1.6646010
H	6.0445780	3.6434220	1.2880640	6.0615390	3.6543320	1.2506010
H	6.8001630	4.0966650	-1.0296280	6.8070150	4.0796390	-1.0755450
H	5.5033520	3.2170460	-2.9498330	5.4979460	3.1807760	-2.9789450
H	3.4398690	1.8769460	-2.5404680	3.4348650	1.8505540	-2.5441040
H	-3.4398690	1.8769470	2.5404670	-3.4348570	1.8505580	2.5441030
H	-3.9819190	2.2988770	-1.6765330	-3.9985430	2.3201620	-1.6645990
H	-6.0445700	3.6434320	-1.2880660	-6.0615370	3.6543410	-1.2505950
H	-6.8001570	4.0966740	1.0296260	-6.8070070	4.0796470	1.0755540
H	-5.5033500	3.2170510	2.9498310	-5.4979360	3.1807820	2.9789500
C	-0.6546000	-3.1488900	-1.3603890	-0.6370840	-3.1359980	-1.3724870
C	0.6545960	-3.1488900	1.3603850	0.6370790	-3.1359980	1.3724850
H	0.3990780	-2.9320220	-1.4488730	0.4166300	-2.9152830	-1.4487430
H	-0.3990820	-2.9320210	1.4488660	-0.4166350	-2.9152810	1.4487390
C	-1.2038760	-4.3226640	-1.7613190	-1.1797110	-4.3055800	-1.7892230
C	1.2038700	-4.3226640	1.7613170	1.1797040	-4.3055800	1.7892230
H	-0.7078510	-5.1756900	-2.1965650	-0.6799800	-5.1542540	-2.2286120
H	0.7078430	-5.1756900	2.1965630	0.6799700	-5.1542530	2.2286120
S	-2.9377800	-4.3818340	-1.4976720	-2.9172660	-4.3660940	-1.5442620
S	2.9377740	-4.3818350	1.4976740	2.9172580	-4.3660970	1.5442640
